# Reproductive Allocation of the Habitat‐Forming Intertidal Macroalga *Ascophyllum nodosum* Decreases at Its Northern Distribution Edge

**DOI:** 10.1002/ece3.72141

**Published:** 2025-09-23

**Authors:** Constança Albuquerque, Birgit Olesen, Núria Marbà, Dorte Krause‐Jensen

**Affiliations:** ^1^ Department of Ecoscience Aarhus University Aarhus Denmark; ^2^ Department of Biology Aarhus University Aarhus Denmark; ^3^ Department of Global Change Research IMEDEA (CSIC‐UIB) Esporles (Illes Balears) Spain; ^4^ Arctic Research Centre Aarhus University Aarhus Denmark

**Keywords:** Arctic ecology, *Ascophyllum nodosum*, Fucoids, latitude gradient, phenology, reproductive ecology, temperature

## Abstract

The habitat‐forming intertidal brown alga *Ascophyllum nodosum* has its colder northern distribution limit at 69°N in Disko Bay, Greenland. Its reproductive effort has never been assessed there despite expected northward expansion with climate change. We analyzed reproductive allocation and phenology at the northern distribution edge and across the geographical distribution range through field studies at three Greenland sites and one Danish site, supplemented with a literature survey. Because *Ascophyllum* is long‐lived and forms annual segments through apical growth, old shoots sampled in the reproductive season revealed receptacle formation with segment age, from the tip to the base of the shoots. We confirmed the fertility of the northernmost populations, as zygotes formed from gametes. We found a consistent pattern of receptacle formation with larger receptacles closer to the canopy top and receptacle abundance following a quadratic relationship with segment age. *Ascophyllum*'s reproductive allocation constitutes 33%–39% of its annual production in the Disko Bay and increases towards southern, warmer latitudes. Reproductive phenology also varies significantly with latitude and temperature, showing a 4.5‐day delay in the reproduction peak for every degree northward and a 14‐day delay with every 1°C decrease in temperature. The carbon flux released from the reproductive structures to the surrounding Arctic ecosystem at the end of the reproductive season was significant, amounting to 212–827 g C m^−2^ year^−1^ in Greenland, which should be considered in future productivity assessments. Synthesis: Our results indicate different life‐history strategies at the opposing distribution edges and stress the importance of temperature as a regulator of *Ascophyllum* reproduction. Arctic warming will likely enhance reproductive output and stimulate an earlier onset of reproduction. Furthermore, the significant contribution from sexual reproduction to the annual carbon production (26%–41% in Greenland) highlighted its importance to the Arctic detrital community.

## Graphical abstract

*Ascophyllum nodosum* is predicted to expand its northern distribution in the sub‐Arctic, but its reproduction has never been assessed at such high latitudes. This study analyzed the reproductive allocation and phenology of *A. nodosum* at its northern distribution limit in Greenland in comparison with populations across its geographical distribution and along latitudinal and temperature gradients. Results revealed varying life‐history strategies at the opposing distribution edges and suggest an increased reproductive allocation and earlier onset of the reproductive season at the northern limit with global warming.

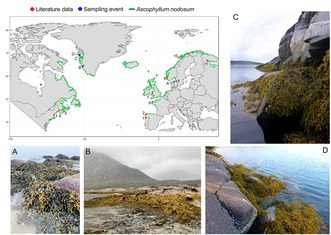

## 1 Introduction

Fucoids are brown canopy‐forming macroalgae that support diverse coastal habitats by providing a physical and functional structure where communities feed, seek shelter, and reproduce (Hawkins et al. 2019; Schmidt et al. 2011). Despite their important role as foundation species in global intertidal areas, fucoids have been systematically “forgotten” (Thomsen et al. 2024). *Ascophyllum nodosum* (Linnaeus) Le Jolis is a conspicuous fucoid that forms highly productive marine “forests” in the North Atlantic Ocean (Cousens 1984) and contributes significantly to carbon and nitrogen cycling (e.g., Duarte et al. 2005; Krause‐Jensen and Duarte 2016; Schmidt et al. 2011). Its large canopy can buffer extreme temperatures and pH fluctuations, and reduce desiccation, facilitating the settlement of macroalgal‐associated species in the otherwise adverse intertidal environment, such as that of the sub‐arctic (Ørberg et al. 2018; Wahl et al. 2018; Sejr et al. 2021).

As the Arctic region is warming at a pace almost four times faster than the rest of the world (Lee et al. 2023; Rantanen et al. 2022; Stroeve et al. 2012), habitats suitable for macroalgae will likely expand in the 21st Century (Assis et al. 2022; Müller et al. 2009; Neiva et al. 2016). Increasing temperatures stimulate the growth of the dominant Arctic algal species, which have temperature optima above current temperatures (Müller et al. 2009), and the associated reduction in sea ice cover expands available habitats (Krause‐Jensen et al. 2012; Marbà et al. 2017; Scherrer et al. 2019). There are already signs of increasing trends for macroalgae in the Arctic (Krause‐Jensen et al. 2020) including that of *A. nodosum* vegetative growth at the northern distribution limit (Marbà et al. 2017). However, changes in species distribution depend on reproductive capacity, which affects the ability to proliferate and survive, often limiting distribution boundaries (Van Den Hoek 1982). In contrast to the northern distribution limit, warmer temperatures threaten *Fucales* species at the rear edge (Álvarez‐Losada et al. 2020; Hernández et al. 2023; Lima et al. 2007), for example, due to impaired reproductive capacity (Ferreira et al. 2015). It is thus a knowledge gap that the limited studies on intertidal macroalgal reproduction are biased towards central range and southern edge populations (Araújo et al. 2015; Bäck et al. 1993; Viejo et al. 2011; Zardi et al. 2015).

One useful framework for studying plant reproduction is the allocation theory, which integrates individual physiology and life‐history theory (Ackerly and Reekie 2005). It provides a quantitative description of the net reproductive output and the investment in reproduction relative to other plant functions. Among species of macroalgae, *A. nodosum* is a good model for studying reproductive allocation because both reproduction and annual vegetative growth can be assessed based on easily distinguishable morphological features when sampled at the appropriate time of year. This is because *A. nodosum* is long‐lived and produces annual bladders through apical growth, resulting in easily distinguishable segments of increasing age from the tip to the base of its long shoots (Marbà et al. 2017; Pereira et al. 2020). Additionally, as receptacles serve the single purpose of reproducing and are shed shortly after, resources invested in sexual reproduction can be readily assessed.

In the case of *A. nodosum* , four studies have quantified reproductive allocation at the central distribution range and one at the southern distribution limit (Åberg 1996; Araújo et al. 2015; Cousens 1986; Mathieson and Guo 1992). Based on these studies, *A. nodosum* typically presents prolific reproduction, with reproductive allocation exceeding 50%. These studies have mainly focused on local and regional‐scale variations and the effect of environmental stressors, such as wave exposure (Cousens 1986; Mathieson and Guo 1992; Vadas et al. 2004), salinity (Mathieson and Guo 1992), and ice scouring (Åberg 1996; Mathieson et al. 1982). The lack of common trends suggests that *A. nodosum* reproductive allocation is highly variable at a small scale and does not necessarily depend linearly on local‐scale environmental factors (Cousens 1986). Large‐scale patterns, however, have the potential to reveal responses to, for example, temperature gradients and, in a climatic space‐for‐time approach (Fukami and Wardle 2005; Pickett 1989), inform about potential future effects of global warming.

So far, only one study has addressed *A. nodosum* reproduction at the scale of the geographical distribution, reporting different life‐history strategies of the southern edge population in Portugal compared to central range populations in France (Araújo et al. 2015). The southernmost population exhibited increased reproductive allocation at the expense of a decreased phlorotannin content, a substance that protects against predation and UV radiation. The authors argued that this was a response to the more stressful conditions at the distribution limits, since seagrass and seaweed often show higher reproductive investment in moderately stressful environments (Dethier et al. 2005; Marbà and Duarte 1995; Meling‐López and Ibarra‐Obando 1999; Phillips and Backman 1983; Robertson and Mann 1984). Whether stressful conditions in the Arctic, such as occasional ice‐scouring, low temperature, and winter darkness, also lead to increased reproductive allocation at the northern distribution edge is unknown.

Latitudinal patterns in reproductive phenology have also been identified for other marine macroalgae and plants extending to the Arctic region. For example, the seagrass *Zostera marina* shows a delay in reproductive phenology towards northern, colder locations (Blok et al. 2018). This is a typical response among macrophytes resulting from latitudinal gradients in temperature and light availability, particularly photoperiod, that is, the light hours in a day (Gorter 1965; Harder 1948). Among fucoid species ( *A. nodosum* , *Fucus serratus* , *Fucus spiralis* , and *Fucus vesiculosus* ) observations suggest an analogous pattern (Fredriksen 1990). In *A. nodosum* , specific photoperiods trigger the differentiation of lateral branches into receptacles (Terry and Moss 1980), while water temperature influences the timing of gamete release (Bacon and Vadas 1991). Consequently, its peak of reproduction occurs in a narrow time window with a seasonality that can vary slightly between years in response to the climate (Åberg 1996; Åberg and Pavia 1997; Mathieson et al. 1976), and at different latitudes (Araújo et al. 2015; Fredriksen 1990). Although no study has examined *A. nodosum* reproductive phenology globally, evidence thus suggests a delayed reproductive phenology towards higher latitudes (Araújo et al. 2015; Fredriksen 1990).

This study aimed to characterize the reproduction of *Ascophyllum nodosum* at and near its northern distribution limit in Greenland and along its broader geographical distribution range. Our analysis covered three scales: (1) the shoot level, to understand patterns of receptacle formation; (2) the population level, to quantify reproductive production and allocation of the target populations; and (3) the species' geographical distribution range, to identify patterns along latitude and temperature gradients. The wide latitude range of *A. nodosum* , from Portugal to Disko Island in Greenland, and the White Sea in Russia, provides exceptional conditions for studying large‐scale geographical patterns in intertidal macroalgae (Pedersen 2022; Schmidt et al. 2011). Through a space‐for‐time lens, these patterns have the potential to elucidate *A. nodosum* 's response to climate change and potential northward expansion. We hypothesized that, similarly to the southern edge, stressful environmental conditions at the northern edge of the geographical range lead to an increase in reproductive allocation compared to the central distribution range. An increase in reproductive investment may entail trade‐offs, such as reduced resources available for vegetative growth, potentially resulting in decreased overall performance in the northernmost populations. Further, we hypothesized a delayed timing of reproductive peak towards northern, colder locations.

## 2 Methods

### 2.1 Study Sites

**FIGURE 1:**
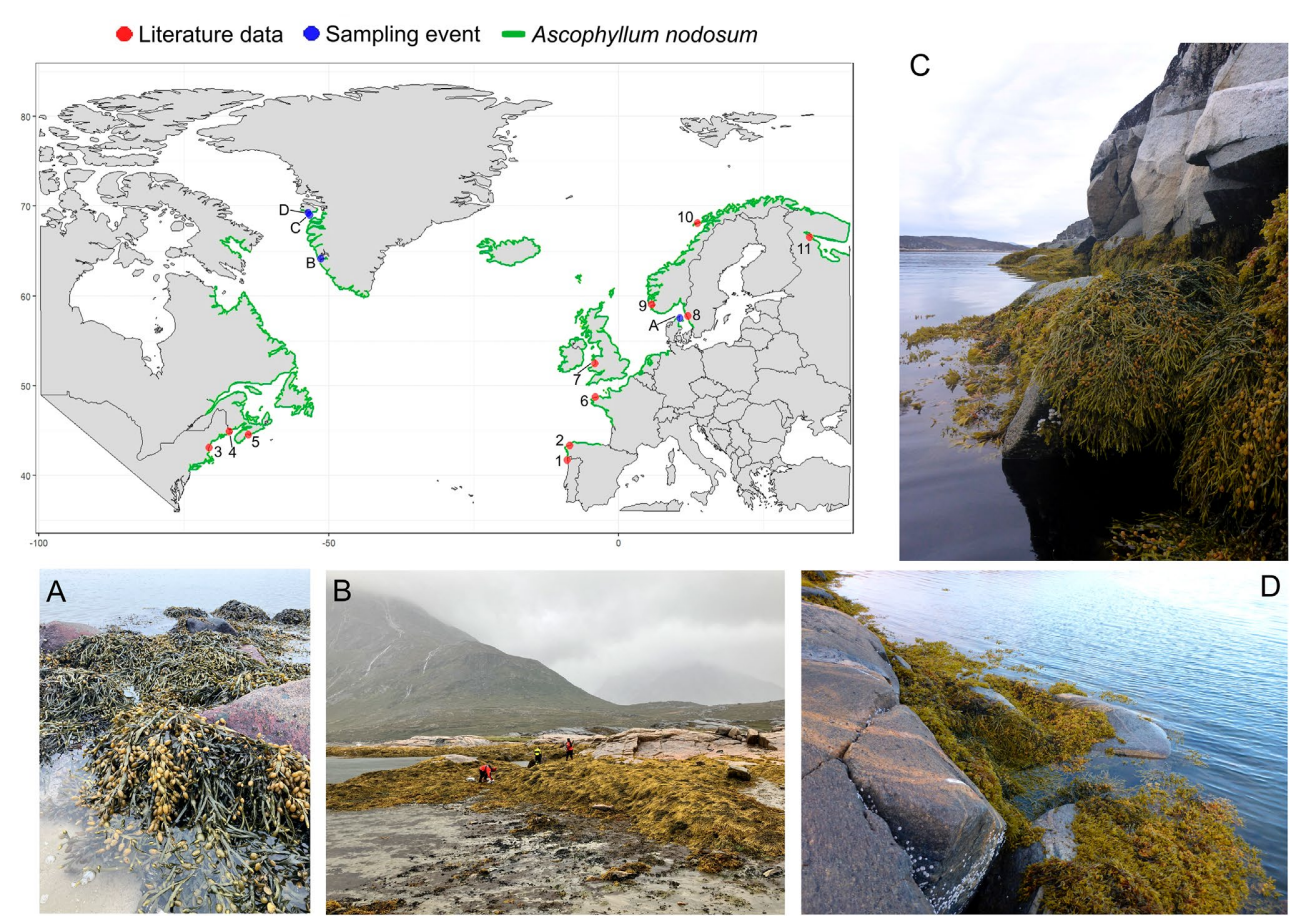
Map showing the location of *Ascophyllum nodosum* study sites sampled for the current study (blue dots, A–D, visualized in photos, details in Tables 1 and 2) and collected from the literature (red dots, 1–11, details in Table 2) as well as the geographical distribution range of *A. nodosum* (green line, redrawn from Pereira et al. 2020). Photos: A‐“Hirsholmene”: Ascophyllum forms dense and narrow populations in the inner bay of a harbor over approximately 150 m, and patches attached to individual rocks in sandy substrate along approximately 70 m of the coastline of the island; (B) “Inner Kobbefjord”: Ascophyllum forms dense, continuous populations extending over more than 1 km of coastline; (C) “Kronprinsen Ejland”: Ascophyllum forms dense populations in a narrow vertical belt, extending over more than 1 km of coastline; (D) “Qeqertarsuaq”: Patchy *A. nodosum* populations in a narrow belt along 20–30 m of coastline intermixed with *Fucus vesiculosus* .

Sampling took place in the mid intertidal zone at three sites on the west coast of Greenland (Figure 1), which are much colder than similar latitudes in the Eastern Atlantic Ocean where the Gulf Stream increases temperatures. We sampled two sites at the northern distribution limit in the Disko Bay: Qeqertarsuaq (Disko Island, 69°24′ N 53°53′ W) representing the northernmost observation of *A. nodosum* in Greenland and Kronprinsen Ejland (69°02′ N 53°32′ W), hereafter named Kronprinsen). The first is a shallow (ca. 1–3 m deep) cove of about 350 m in diameter exposed to a tidal amplitude of up to 3 m (Thyrring et al. 2021, obtained from ocean.dmi.dk), and sheltered from ice scouring by rocky boulders. The latter constitutes a protected rocky channel, with similar tidal ranges. At these sites salinity ranges at least between 29.6 and 33.1 (Krause‐Jensen and Duarte 2016). The third Greenland site is within the inner part of Kobbefjord, in the Nuup Kangerlua fjord system near Nuuk (64°14′ N 51°40′ W), with a tidal amplitude of up to 5 m (Thyrring et al. 2021, obtained from ocean.dmi.dk). It shares some of the characteristics of the northernmost populations, such as year‐round temperatures below the growth optimum of 15°C–25°C (Fortes and Lüning 1980; Keser et al. 2005; Strömgren 1983; Pereira et al. 2025), and the presence of sea ice. The inner Kobbefjord also occasionally experiences reduced salinity due to ice melt and glacier runoff, ranging at least between 28.9 and 31.7 (Krause‐Jensen and Duarte 2016; Rysgaard et al. 2003). We sampled an additional site centrally in the geographical distribution range, on the island of Hirsholmene, Denmark (57°45′ N 10°62′ E) (Figure 1). This site is located within the inner bay of the harbor and experiences an average tidal range of ~0.30 m (based on nearest reference point: Frederikshavn). The average annual salinity is 26.2 (2020–2024, data from nearest monitoring stations of the Danish National Monitoring Program (NOVANA)). All study sites present relatively sheltered conditions. Further sites with information on *A. nodosum* reproduction were compiled from the literature (Figure 1).

### 2.2 Data Collection

Sampling for annual reproductive biomass occurred at the peak of the reproduction season, and for vegetative growth at the end of the growth season (Table 1 for details). At the northernmost sites (Kronprinsen, Qeqertarsuaq, 69° N) these two timings coincide in late August, which allowed for quantification of both annual reproductive and vegetative production from the same sampling (Marbà et al. 2017). Previous observations at these sites indicated that the reproduction season occurs in August (authors' unpublished data), a pattern confirmed during our sampling in late August, when abundant mature receptacles were found. For Kobbefjord, Nuuk (64° N), vegetative production was quantified through sampling in early September, while reproduction was quantified through samples collected in early July. Shoots sampled in July had a high abundance of mature receptacles, whereas only a few remained in September, which confirmed the reproduction peak to occur in July. At Hirsholmene (57° N), sampling for vegetative growth took place in November, while reproduction was measured during the reproduction peak in April. This timing was identified following our own visual inspection in November, March, and April, and observations from the literature (Nielsen et al. 2022).

**TABLE 1:** Location of *Ascophyllum nodosum* sampling sites in this study; sampling dates; standing biomass in August/September for the Greenland populations and November for the Hirsholmene population, Sampling at Hirsholmene targeted areas of maximum biomass density. Average and standard error are presented, *n* = 5.

Site	Geographic coordinates	Sampling date (dd/mm/yy)	Standing biomass (g DW m^−2^)
Denmark			
(A) Hirsholmene	57°45′ N 10°62′ E	6/11/2023 6/04/2024^a^	8683 ± 2010
West Greenland			
(B) Kobbefjord	64°14′ N 51°40′ W	3/07/2023^a^ 5/09/2023	6860 ± 888
Disko Bay			
(C) Kronprinsens Ejland	69°02′ N 53°32′ W	30/08/2023^a^	—
(D) Qeqertarsuaq	69°24′ N 53°53′ W	26/08/2023^a^	6522 ± 1751

^a^ Approximate time of reproduction peak (±15 days).

#### 2.2.1 Sampling of Individual Shoots

**FIGURE 2:**
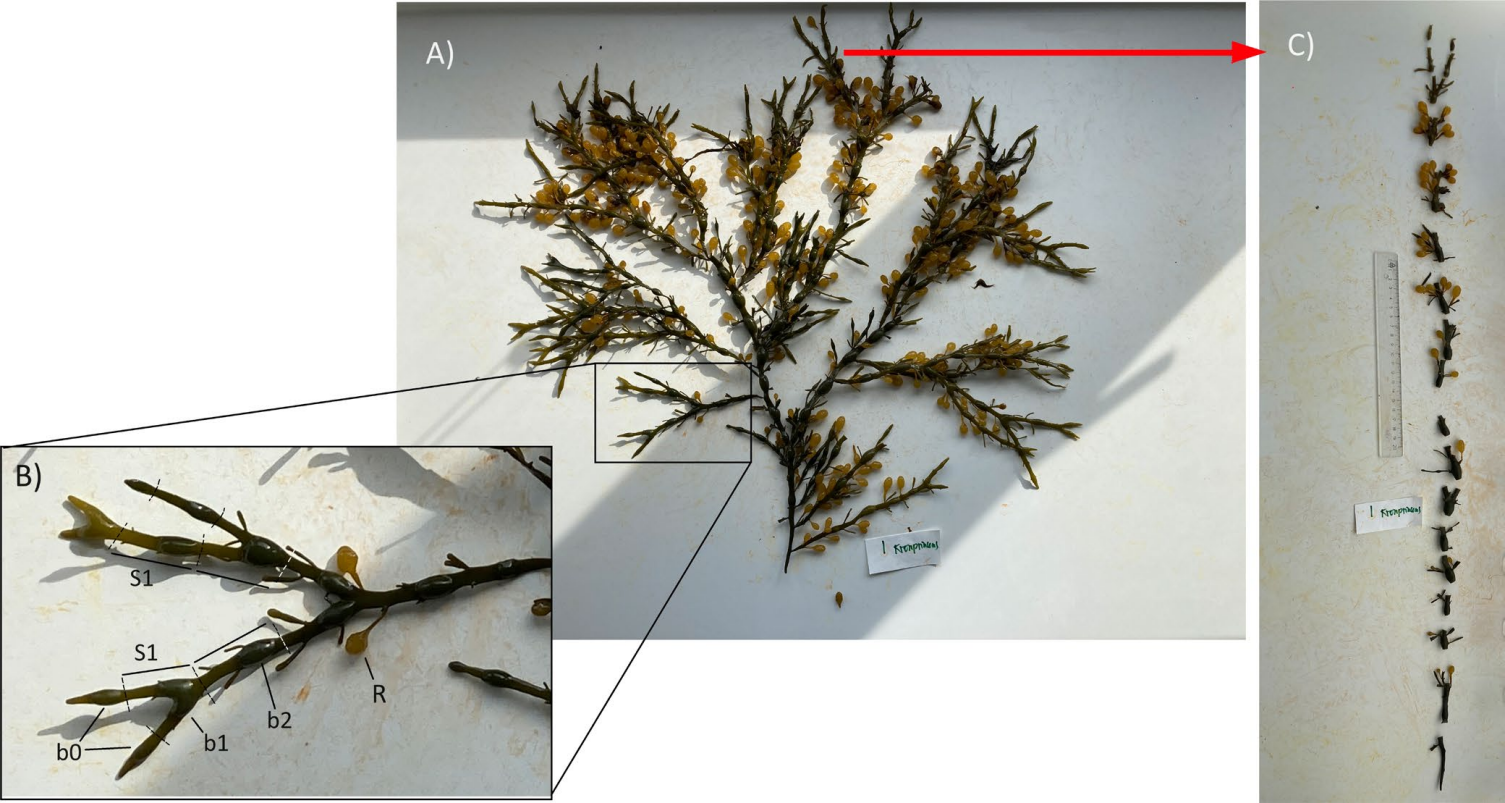
(A) Long shoot of *Ascophyllum nodosum* , from Kronprinsens Ejland (minimum age estimate of 18 years). Red arrow links to the tip of the longest primary branch. (B) Detail of *A. nodosum* tip section, that includes the three youngest segments; S1: 1‐year‐old segment; b0: Youngest bladder; b1: Second youngest bladder (1‐year‐old); b2: Third youngest bladder (2 years old); R: Receptacle. (C) Longest primary branch from the shoot presented in image (A). Branch fragmented in age segments (18 total).

In this study, “shoot” refers to the longest, unbroken primary shoot, preferably originating directly from the holdfast, and respective lateral shoots (Figure 2). This definition does not encompass the entire frond originating from one holdfast, but rather a part of one individual. In the rare cases when the apical meristem of the primary shoot was broken, we used the longest unbroken lateral shoot instead. At each site, 10–14 unbroken shoots were randomly collected at the mid‐intertidal range, cut by the holdfast. The longest and, hence, oldest shoots were targeted. In Greenland, where individuals grow older than at Hirsholmene, we aimed for shoots with at least 10 bladders, corresponding to at least 10 consecutive years of growth. In contrast, the longest shoots at Hirsholmene had a maximum of only five bladders, so we targeted those. In Greenland, we sampled apical segments from 20 additional individuals, resulting in apical meristems from a total of 30 individuals. In Hirsholmene, 20 individuals were assessed for apical growth. At all sites, shoots originated from different holdfasts to ensure selection of distinct individuals. Samples were placed immediately inside plastic bags and preserved at 5°C or frozen, depending on whether they were analyzed in the laboratory right after sampling or later on.

### 2.3 Fertility

This species is dioecious, and sexes can be identified by examining sections of the receptacles under the microscope. To assess fertility at the northernmost distribution limit, mature male and female receptacles were collected from Qeqertarsuaq and Kronprinsens Ejland. They were rinsed with freshwater, placed on moistened blotting paper overnight at 8°C, and subsequently placed in seawater in a petri dish. After 2–3 days, the samples were observed under the microscope using microscopic slides to check for the formation of zygotes.

### 2.4 Receptacle Formation at the Shoot Level

Receptacle formation was assessed for each site individually, by selecting the primary axis of each of the 10 shoots, that is, the longest intact branch that did not originate from a lateral pit (Figure 2C). We then fractioned it into age segments by cutting below each bladder and counted all the receptacles attached to each segment. Since most shoots were not older than 12 years, we limited the analysis of segments to that age. Receptacles from the 10 shoots were pooled by segment age, dried at 60°C for at least 24 h until constant weight, and weighed to obtain an average receptacle weight per segment age.

### 2.5 Vegetative Growth and Annual Reproductive Allocation

#### 2.5.1 Annual Vegetative Growth (annV)

Vegetative growth occurs at the apical meristem and can be estimated by measuring distinguishable annual segments, consisting of one air bladder (vesicle) and a consecutive branch segment (Figure 2). We estimated annual growth based on the newest completely formed segment, that is, segment year 1 (S1 in Figure 2B). Segments 1 from primary branches were selected, dried at 60°C for at least 24 h, and weighed. This segment represents a minimum estimate of annual growth, since the segment may continue to accumulate biomass for a few years (up to 5 years at temperate locations, Lauzon‐Guay et al. 2022), although growth is primarily apical. To estimate the total annual growth of each shoot, the average annual growth of segments S1 can be multiplied by the total number of segments S1 per shoot (counts of bladder b1, at the base of S1), or by the number of apical segments not fully formed yet (counts of bladder b0, just below the apical meristems). The shoots of *A. nodosum* tend to divide dichotomously, leading to a progressive increase in the number of apical tips each year, unless branches are cut and dislodged. Therefore, counts of bladder b1 may lead to an underestimate, while counts of bladder b0 likely lead to an overestimation of the apical tips formed during 1 year period. To meet the best possible compromise, annual vegetative growth (annV) per shoot was calculated as:

(1) $$\text{annV}\;\left(g\right)=\text{mean}\;S1\;\mathrm{DW}\;\left(g\right)\times n$$

where *n* denotes the number of tips per shoot, calculated as the average of the two estimates (b0 and b1 counts), and S1 DW is the dry weight of segment 1. The counts of b0 and b1 were performed by analyzing individual pictures of the shoots using the software ImageJ/Fiji. To compare vegetative production across populations, we also estimated the shoot turnover rate as the ratio between annV and the dry weight of the entire shoot without receptacles (*V*):

(2) $$\text{Turnover rate}=\frac{\text{annV}}{V}$$

We estimated annual reproductive allocation (annRA) (Equation 3) and reproductive effort (RE) (Equation 4) as:

(3) $$\text{annRA}=\frac{R}{\left(R+\text{annV}\right)}$$

(4) $$\mathrm{RE}=\frac{R}{V}$$

where R is the annual reproductive biomass (see below), annV is the annual vegetative growth, and *V* is the vegetative biomass per shoot.

For perennial plants and macroalgae, annRA gives a more reliable comparison across different individuals and species, as it excludes older vegetative biomass, which may vary significantly (Åberg 1996). RE was also estimated to allow for direct comparisons with estimates from other studies on *A. nodosum* (Mathieson and Guo 1992). Given the ambiguity of the term reproductive effort, which has been used with different meanings historically, we use it here solely as a descriptive measure of reproductive output, and not as an assessment of reproductive cost (Ackerly and Reekie 2005; Karlsson and Méndez 2005).

#### 2.5.2 Annual Reproductive Biomass (R)

Because the reproductive peak of *A. nodosum* occurs once annually within a very short period (except at the southern distribution edge), annual reproductive biomass can be estimated from a single sampling during the reproduction peak, just prior to gamete release. To estimate R, the receptacles of each of the 10 to 14 shoots were counted, dried at 60°C for at least 24 h, and weighed.

#### 2.5.3 Annual Carbon Production

Annual reproductive biomass and vegetative growth were also expressed in carbon (C) units by analyzing the C‐content of dry, ground biomass using a CN analyzer (Vario EL Cube, Elementar, Langenselbold, Germany). Vegetative C‐content was measured in segment 1 (S1); and reproductive C‐content was measured in receptacles collected along the branch at segment ages 2, 5, and 8. The mean C‐content of S1 (*n* = 3) was multiplied by the annual vegetative growth (annV) while the mean C‐content of the receptacles (*n* = 3 for each segment age) was multiplied by the annual reproductive biomass (R) to express both vegetative and reproductive productivity in C‐units. Following Equation (3) it was then possible to estimate annRA in C‐units.

#### 2.5.4 Upscaling to Annual Productivity per Area

To estimate the C‐flux per square meter, estimates of the standing biomass per area were multiplied by the fractions corresponding to reproductive biomass and annual vegetative growth. These fractions are represented by the reproductive effort (RE, Equation 4) and shoot turnover rate (annV/V, Equation 2). Standing biomass was measured in Kobbefjord and Qeqertarsuaq in 2010 and 2009, respectively (authors' unpublished data) and are expected to have remained stable as shown for the *A. nodosum* population in Kobbefjord (author's unpublished data). In Hirsholmene, standing biomass was measured using five 20 × 20 cm quadrats, which were non‐randomly placed in the intertidal area, specifically targeting vegetation on scattered boulders. These measurements were taken in November 2023. Due to logistic constraints, we could not obtain the standing biomass measurements for Kronprinsen, so estimates of productivity per area are omitted for this site.

### 2.6 Literature Survey

In addition to our own sampling, we searched the existing literature for data on the reproductive allocation of *Ascophyllum nodosum* , and found five studies, representing six different regions—north Portugal, New England (USA), Maine (USA), Nova Scotia (Canada), northwest France, and southwest Sweden. Three of these studies calculated annRA using the formula (Equation 3) presented above. Two of them estimated annRA based on individual shoots, while the third used standing crop estimates (m^−2^) (see Table 2 for detailed information). The study by Vadas et al. (2004) did not directly present an estimate of annRA, but it was possible to calculate it (Equation 3) using their data on standing crop reproductive and vegetative productivity. Since the reproduction peak in that region (Maine, USA) takes place in the spring, we used data collected during that period. We selected the unadjusted data (without seasonal biomass loss adjustment), as it better represents the data type used in the other studies. Finally, the study from Mathieson and Guo (1992) in New England only presented estimates of reproductive effort, not annRA, so it was excluded from the regression analysis. At the end, a total of four studies in addition to ours provided data of annRA from nine regions covering a 27° latitude range from the southern distribution edge in Portugal at 42° N to the northern edge at 69° N (Table 2). The Portuguese dataset was based on one population only, that is, the southernmost population of *A. nodosum* (Araújo et al. 2015) (Table 2). In France (Araújo et al. 2015) and Sweden (Åberg 1996) the value provided in the study refers to an average of two populations that served as replicates. In France, the two populations are in the same region (Roscoff and Santec, Brittany), separated by around 4 km; while in Sweden the populations were in different regions (Tjärnö and Göteborg) but were not significantly different, and were therefore treated as replicates in the original publication. In Nova Scotia (Cousens 1986), there were originally nine different populations distancing around 80 km between each other, which we grouped in three regions (Inner, South and North Nova Scotia), and the respective means were used in this analysis, making a total of 11 data points altogether. The data from Maine (Vadas et al. 2004) included four different populations located within the same embayment system.

**TABLE 2:** Location, sampling year (when available) and data source of *Ascophyllum nodosum* studies included in the latitudinal comparison of reproductive allocation and time of reproductive peak.

Site	Geographic coordinates	Data source (author)	Sampling date (year)	Measure of reproductive allocation	annRA (ratio year^−1^)
(1) Portugal	42 N 9 W	Araújo et al. (2015)	—	annRA = *R*/(*R* + annV)	0.76
Spain (2) Galícia	43 N 8 W	Viana et al. (2014)	2011	—	—
USA (3) New England	43 N 71 W	Mathieson and Guo (1992)	1988–1991	RE = *R*/V	0.53 (*)
(4) Maine	45 N 67 W	Vadas et al. (2004)	1995–1996	annRA = *R*/(annV)	0.61 (**) (***)
Canada (5) Nova Scotia	44 N 64 W	Cousens (1986)	1979–1980	annRA = *R*/(*R* + annV)	0.56 (***)
(6) France	48 N 4 W	Araújo et al. (2015)	—	annRA = *R*/(*R* + annV)	0.54
UK (7) Wales	52 N 4 W	Lewis (2020)	2018–2019	—	—
(8) Sweden	58/59 N 11/12 E	Åberg (1996)	1985–1986	annRA = *R*/(*R* + annV)	0.53
Norway (9) Stavanger	59 N 6 E	Fredriksen (1990)	—	—	—
(10) Lofoten	68 N 14 E	Fredriksen (1990)	—	—	—
Russia (11) White Sea	67 N 33 E	Maximova and Sazhin (2010)	2005	—	—
Denmark (A) Hirsholmene	57°45′ N 10°62′ E	This study	2024	annRA = *R*/(*R* + annV)	0.51
Greenland (B) Kobbefjord	64°14′ N 51°40′ W	This study	2023	annRA = *R*/(*R* + annV)	0.50
(C) Kronprinsen Ejland	69°24′ N 53°53′ W	This study	2023	annRA = *R*/(*R* + annV)	0.33
(D) Qeqertarsuaq	69°02′ N 53°32′ W	This study	2023	annRA = *R*/(*R* + annV)	0.39

*Note:* For each source, the formula used to estimate reproductive allocation is given. Specifications: (*) Only RE was provided in this study, not allowing us to calculate the annRA and the direct comparison with the remaining studies; (**) AnnRA was calculated by us (annRA = *R*/(*R* + annV)), based on reproductive and vegetative productivity estimates provided in the source; (***) Measures correspond to standing crop estimates (m^−2^), and not individual shoot estimates.

Abbreviations: AnnRA, annual reproductive allocation; annV, vegetative biomass produced annually; R, reproductive biomass; RE, reproductive effort; *V*, total vegetative biomass.

In addition to the five studies above, we gathered data on the timing of reproduction from four more studies, resulting in a total of 11 different regions, spanning the entire distribution range (Figure 1). When the term “reproduction peak” was not explicitly mentioned, the following terms were considered synonymous: “peak of gamete release” (Araújo et al. 2015), “mature receptacles” (Viana et al. 2014), “prior to gamete release” (Åberg 1996; Vadas et al. 2004), “receptacles at their largest” (Cousens 1986), “spawning” (Lewis 2020), and “active fruiting” (Maximova and Sazhin 2010). Therefore, we defined the reproduction peak as the period when gametes are mature encompassing the time just prior to and during the initial phase of gamete release. The timing of reproduction peak was estimated in approximate Julian days (JD) (±15), by assigning the 15th day of the month of the reproduction peak to each respective site.

### 2.7 Latitude and Temperature Data

To analyze large‐scale patterns in reproductive allocation and phenology across the distribution range, we correlated this dataset with latitude and mean sea surface temperature (SST). While latitude is highly correlated with key environmental variables such as photoperiod and SST, the latter may also vary with ocean currents and local hydrographical conditions. Thus, testing correlations with both latitude and SST allowed us to better capture the main environmental variations that explain possible large‐scale patterns.

When the surveyed literature did not provide exact coordinates for the compiled study sites, approximate latitudes were obtained from Google Maps, based on the descriptions and/or maps provided. SST for all locations was retrieved from the open‐source Bio‐ORACLE v3 database, which offers worldwide environmental data layers with a 0.05° resolution for marine ecological modeling (Assis et al. 2024). Data layers were retrieved using the biooracler package in R version 4.3.2, and managed with the terra package (Hijmans 2025) in RStudio 2023.12.1 + 402 “Ocean Storm”. When SST data was unavailable for the exact site coordinates, we estimated values from the nearest available location, usually offshore from the sampling site. The data available represented 10‐year averages for the decades 2000–2010 and 2010–2020, which were averaged into a long‐term mean SST for the period 2000–2020. We aimed for the longest possible long‐term estimate to ensure a general and robust characterization of each region, disregarding the influence of annual variability.

### 2.8 Statistical Analysis

The effect of segment age on the number and size of receptacles was assessed using linear models (LM), produced for each site individually. For the number of receptacles, a generalized linear mixed‐effects model (GLMM) with the negative binomial distribution and logarithmic link function was used, supported by the R‐package lme4 (Bates et al. 2015). This model accounted for non‐independent observations, that is, repeated measures along each primary axis, by including primary shoot ID as a random effect. The model accounted for both random intercepts and slopes depending on shoot ID, and fixed effects in this model were age and age^2^. For receptacle size, a LM was used, with segment age as the single predictor. In this model, there were no non‐independent observations, as only the mean receptacle size per segment age per site was tested. Differences among the four sites were tested using one‐way analysis of variance (ANOVA) and means compared using post hoc Tukey's HSD.

Effects of site and vegetative shoot size on the turnover rate, reproductive biomass, reproductive effort, annRA, and carbon productivity per area were tested using LM. A LM was used when the data were parametric (e.g., forannRA in dry weight (g) and C‐units). In these cases, the means were compared with post hoc Tukey's HSD applied to the raw data. Turnover rate, reproductive effort, and reproductive productivity per area did not meet the requirements of normality and homogeneous variances across sites. Therefore, a generalized linear model (GLM) was used, with a Gamma distribution and a logarithmic or a square root link function in the case of reproductive productivity per area (Zuur et al. 2009). Pairwise differences among the sites were then tested on the log‐transformed turnover and reproductive effort, and root‐transformed reproductive carbon productivity per area, using a post hoc Tukey's HSD (Dalgaard 2008). The effect of shoot vegetative size was tested for all variables independently, using LM or GLMM and selecting site as a random effect. Reproductive biomass was the only variable significantly affected by vegetative shoot size (Appendix S1: Figure S1, Table S9).

The relationships between reproductive allocation (annRA) and time of reproductive peak (Julian day) and latitude (degrees N) and SST (°C), respectively, were tested in separate linear regression analyses, since high collinearity between these predictors would be an issue in a multivariate approach (Pearson's *r* = −0.78). The analysis was performed using average values for each region/study, which disregard local scale variability. While this may reduce statistical power, we believe it does not introduce significant biases, as each individual mean was based on larger sample sizes (20–210 replicates among the literature surveyed), obtained through randomized procedures. The regression residuals for time of reproduction peak as a function of SST just met the requirements of normality if using a more conservative alpha value (*p* < 0.01) (Shapiro–Wilk test: *p* = 0.02). Nevertheless, we argue that it is reasonable to opt for a linear regression, since the deviation from normality was not very high. To minimize type 1 error, we also used a more conservative alpha value (*p* < 0.01) to interpret the regression analysis.

Normality and heteroscedasticity were evaluated by visual inspections of Q–Q plots and residual plots. Additionally, normality was confirmed with Shapiro–Wilk test, and homoskedasticity was assessed with Levene test or Goldfeld–Quandt test, for categorical or continuous predictors, respectively. All analyses were performed in R version 4.4.2 (2024‐10‐31 ucrt) “Pile of Leaves,” with the help of software RStudio 2023.12.1 + 402 “Ocean Storm”. In addition to the R‐packages already mentioned, the following also supported our analysis: tidyverse (and associated packages) (Wickham et al. 2019), lubridate (Grolemund and Wickham 2011), car (Fox and Weisberg 2019), lmtest (Zeileis and Hothorn 2002), nlme (Pinheiro et al. 2025), glmmTMB (McGillycuddy et al. 2025), lme4 (Bates et al. 2015), terra (Hijmans 2025), ggplot2 (Wickham 2016), and RColorBrewer (Neuwirth 2022).

## 3 Results

### 3.1 Population Fertility

**FIGURE 3:**
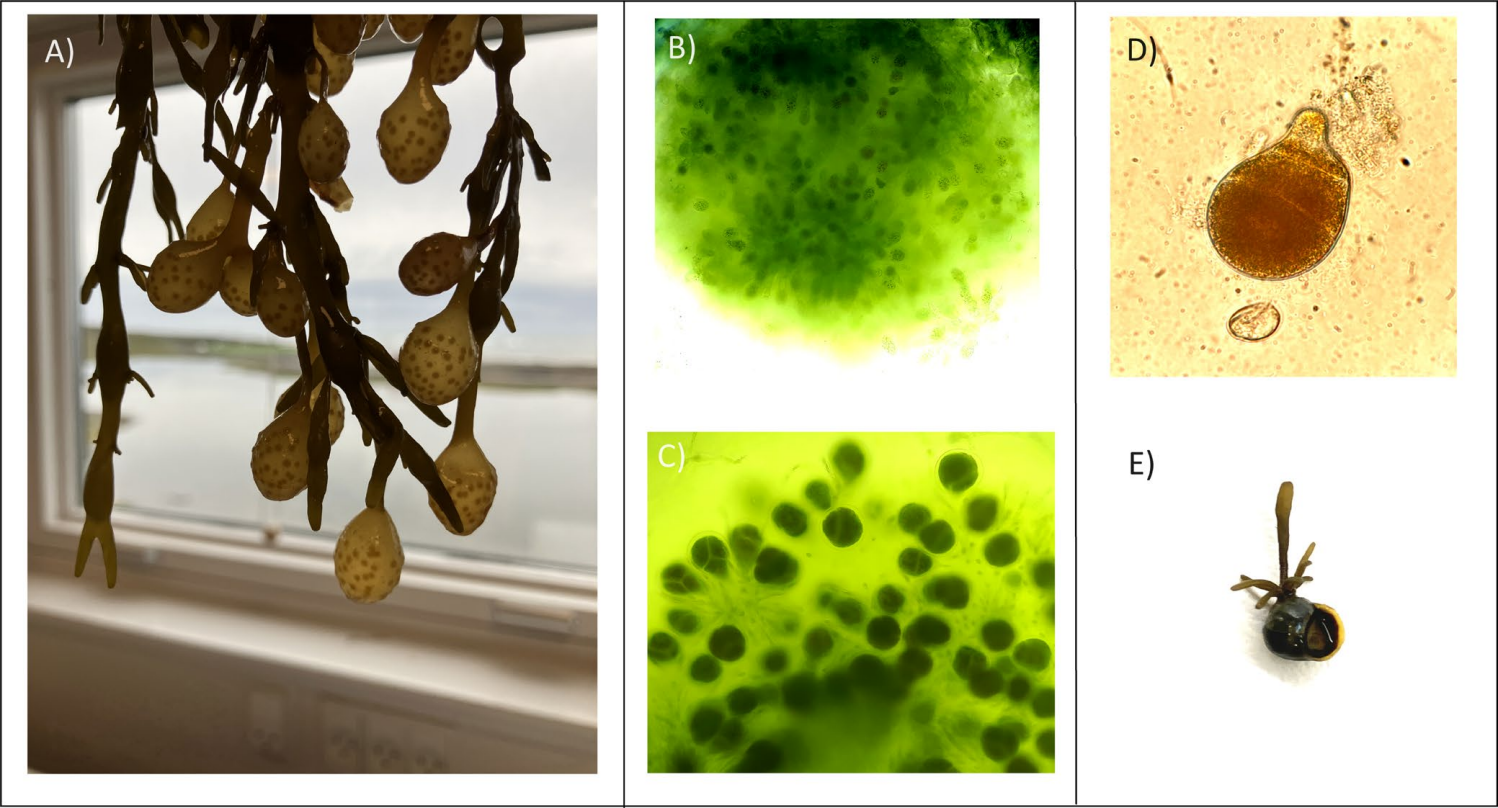
(A) Branches of *Ascophyllum nodosum* with mature receptacles. (B) Section of male conceptacle with antheridia (amplification ×100). (C) Section of female conceptacle and oogonia with eggs (amplification ×100). (D) Zygote of *Ascophyllum nodosum* with four cells (amplification ×100). (E) Germling of *Ascophyllum nodosum* germinated on top of a sea snail.

In all sampled populations, we identified male conceptacles with branched filaments and mature antheridia (Figure 3A,B) as well as female conceptacles with mature oogonia containing four eggs (Figure 3C). The mature receptacles documented that we sampled during the reproduction peak. In Kobbefjord in July and in Hirsholmene in April, a small number of receptacles were at an earlier stage of development, that is, oogonia not yet divided, while in Qeqertarsuaq and Kronprinsen, the oogonia observed had all undergone division. We detected zygotes formed from gametes and juveniles from the two northernmost Greenlandic populations, which confirms fertility at the northern distribution edge (Figure 3D,E).

### 3.2 Receptacle Formation at the Shoot Level

**FIGURE 4:**
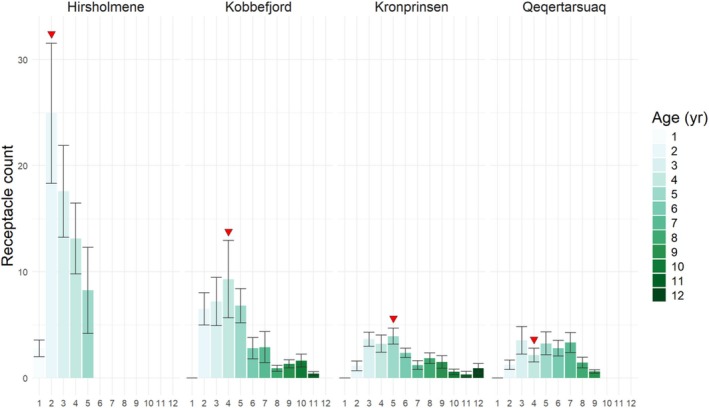
Mean number of receptacles in each annual segment for each of the sampled populations. Segments are identified by age (yr) according to their position on the main axis of the shoot. Black vertical lines indicate the standard error. Red arrowhead indicate the segment age at which 50% of the receptacles (counting from the apical part) were produced. *n* = 10–14.

In Kobbefjord and Hirsholmene, there were no receptacles in the youngest segment (age 0), but all segments aged 1–11 years produced receptacles (Figure 4). In Qeqertarsuaq and Kronprinsen, there were no receptacles in the youngest and second youngest segments (age 0 and 1), but all segments aged 2–12 produced receptacles (Figure 4). The number of receptacles increased from the apical tip up to a maximum and then decreased towards the oldest origin of the shoot, describing a quadratic relationship with segment age (*p* < 0.001; Appendix S1: Table S2). This relationship was significant for all four populations. Most receptacles were produced in the youngest part of the shoot. In Kobbefjord and Qeqertarsuaq, half of all receptacles were produced up to segment age 4 years, and in Kronprinsen, up to segment age 5 years (Figure 4, red arrows). In Hirsholmene, where maximum segment age was only 5 years old, half of the receptacles were produced up to segment age 2 years.

**FIGURE 5:**
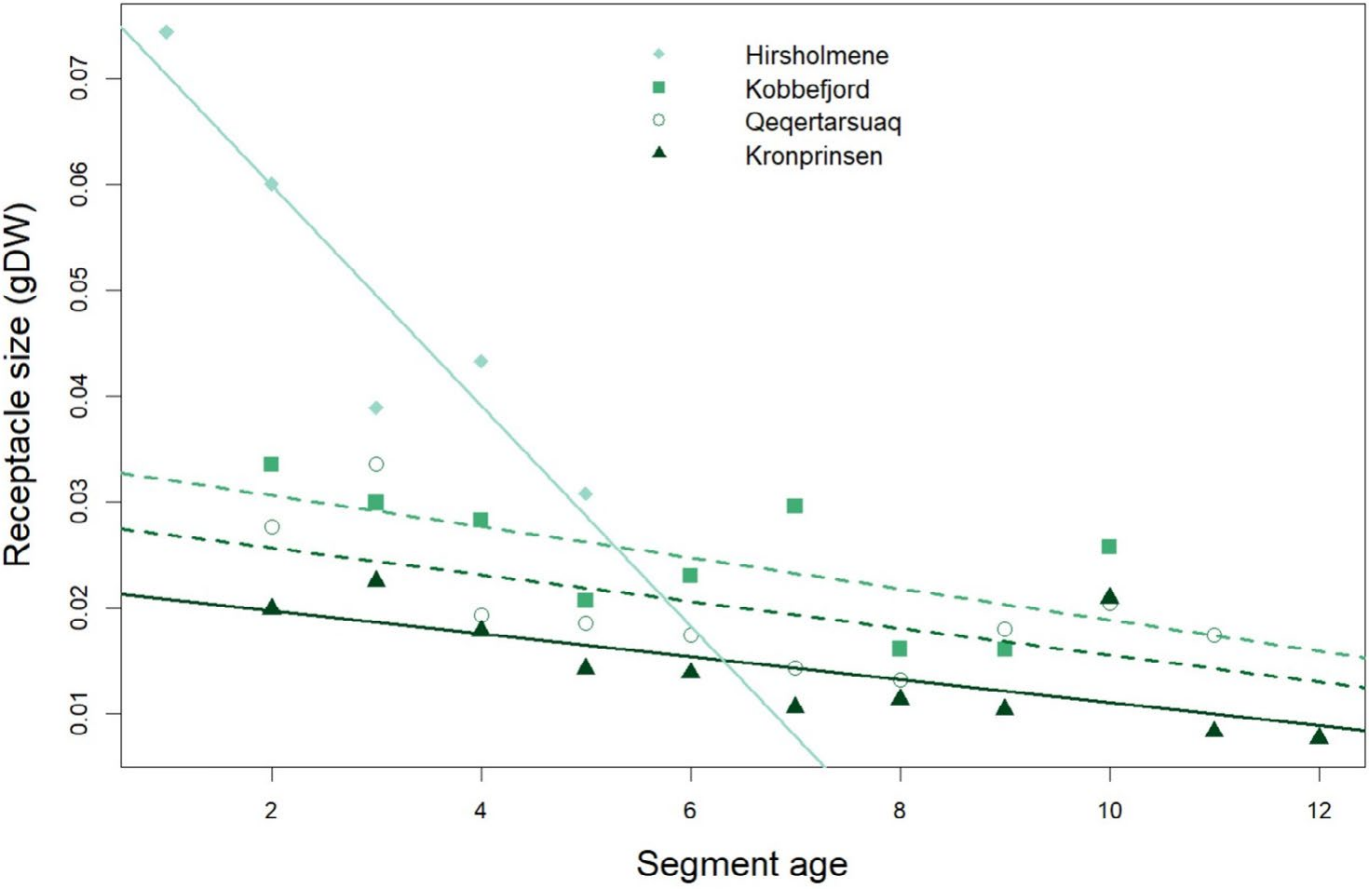
Mean receptacle size (RS) (g DW) as a function of segment age (SA) (Yr). Colored lines indicate respective linear regressions for each sampling site. Dashed lines indicate the regressions that are not statistically significant. (Kobbejord: RS = −0.0015 SA + 0.034; *p* = 0.0596; *R* ^2^ = 0.34; Qeqertarsuaq: RS = −0.0013 SA + 0.028; *p* = 0.0536; *R* ^2^ = 0.31; Kronprinsen: RS = −0.0011 SA + 0.023; *p* = 0.020; *R* ^2^ = 0.41; Hirsholmene: RS = −0.010 SA + 0.081; *p* = 0.0189; *R* ^2^ = 0.84).

The dry mass per receptacle decreased with segment age; the decrease being significant in Kronprinsen and Hirsholmene and marginally significant in Kobbefjord and Qeqertarsuaq (Kobbefjord: *p* = 0.0596; Qeqertarsuaq: *p* = 0.0536; Appendix S1: Table S3) (Figure 5). The linear regressions of size as a function of age show different *y*‐axis intercepts according to the site, reflecting a difference in receptacle size among sites. ANOVA and post hoc Tukey HDS test confirmed that differences in dry mass per receptacle are significant between Kobbefjord and Kronprinsen, and between Hirsholmene and the other three populations (Appendix S1: Table S4). Based on a total of 10 to 14 shoots per site, the average receptacle dry mass was 0.025 g (SE = 0.002) in Kobbefjord, 0.020 g (SE = 0.002) in Qeqertarsuaq, and 0.014 g (SE = 0.002) in Kronprinsen. In Hirsholmene, average receptacle size was 0.050 g (SE = 0.008), twice the size or more compared to the other populations.

### 3.3 Reproductive and Vegetative Productivity

#### 3.3.1 Vegetative Biomass Turnover

**FIGURE 6:**
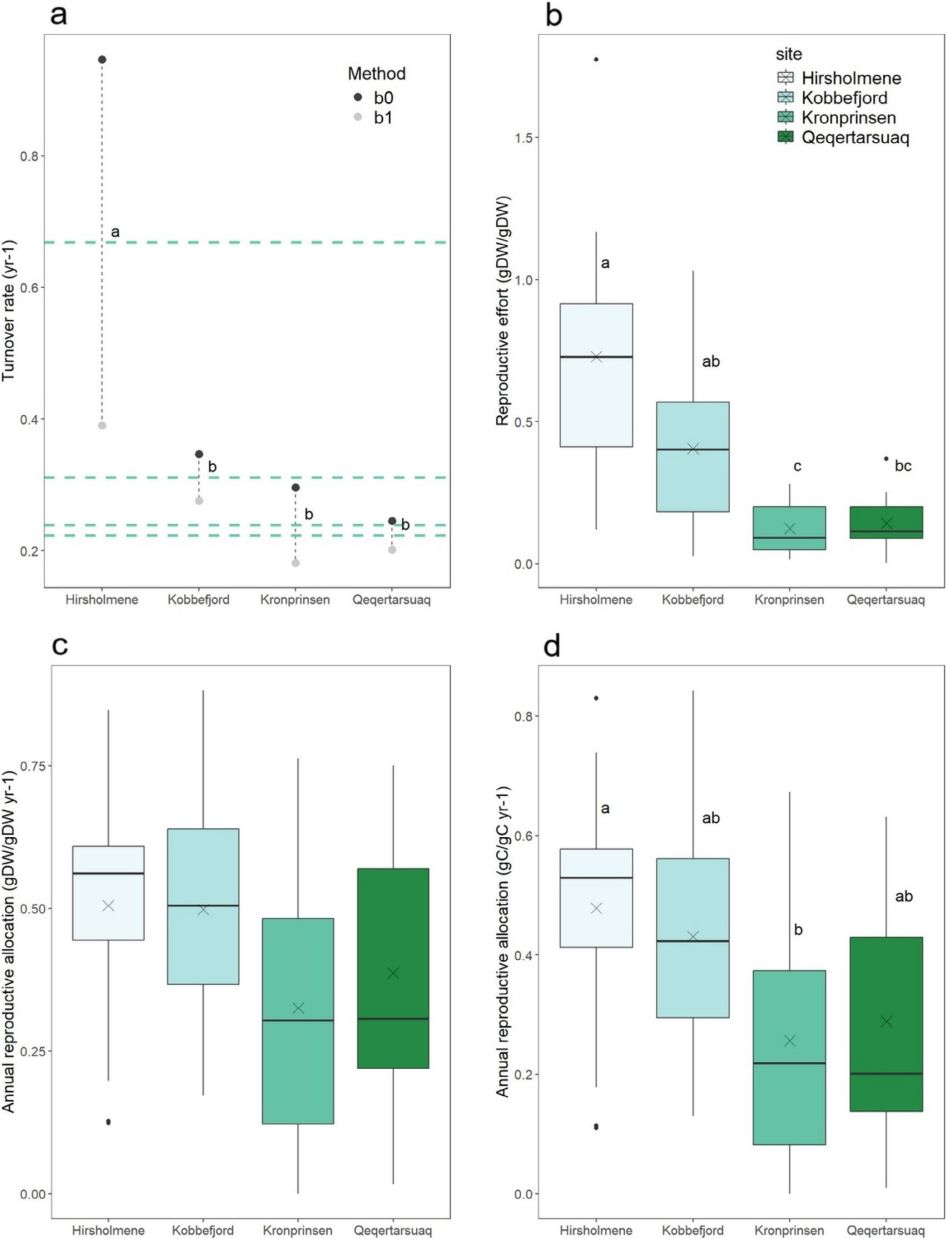
(a) Turnover rate (year^−1^), as the ratio between the annual vegetative production and the total shoot vegetative biomass. The annual vegetative production estimated by multiplying the average segment S1 biomass (g DW) with the number of youngest bladders (b0) per shoot (black symbols) or the number of second youngest bladders (b1) (gray symbols) (Kobbefjord, Qeqertarsuaq, Kronprinsen: *N* = 30; Hirsholmene: *N* = 20). Green dashed line indicates the mean between the two estimates. Letters indicate differences at a level of significance equal to 0.05. (b) Reproductive effort (RE), as the ratio between shoot reproductive production and total shoot vegetative biomass (*n* = 10–14). The boxplot indicates median, first and third quartile (percentile 25th and 75th); Cross indicates the estimated mean. (c) Annual reproductive allocation (annRA), as the ratio between reproductive production and total annual production (reproductive and vegetative) (*n* = 10–14). (d) annRA, as the ratio between reproductive carbon productivity and total annual carbon productivity (*n* = 10–14).

The average biomass turnover rates differed markedly depending on whether annual tip production was estimated based on the number of youngest bladders (b0) or second youngest bladders (b1) (Figure 6a); the average between the two measures was used in all following calculations. However, independently of the method used, the mean turnover rate of vegetative biomass tended to increase from the northernmost locations (Qeqertarsuaq, $\bar{x}$ = 0.24 SE = 0.04 year^−1^ and Kronprinsen, $\bar{x}$ = 0.22 SE = 0.03 year^−1^), towards south Kobbefjord ($\bar{x}$ = 0.31 SE = 0.05 year^−1^) and Hirsholmene ($\bar{x}$ = 0.67 SE = 0.09 year^−1^) (Figure 6a). The effect of site on the mean turnover rate was significant (GLM, *p* < 0.001; Appendix S1: Table S5), with significant differences between Hirsholmene and the Greenlandic populations, but not between Greenland sites (Kobbefjord–Kronprinsen: *p* = 0.54; Kobbefjord–Qeqertarsuaq: *p* = 0.54, post hoc Tukey HSD test). Lack of significant differences between the Greenland populations can derive from the high variability in our samples and small number of replicates (*n* = 10–14).

#### 3.3.2 Reproductive Effort

Variability in reproductive production among shoots was high in all sites; some shoots had numerous receptacles, while others presented almost none. The reproductive effort was higher in Hirsholmene ($\bar{x}$ = 0.73 SE = 0.12) and Inner Kobbefjord ($\bar{x}$ = 0.41 SE = 0.10), compared to Qeqertarsuaq ($\bar{x}$= 0.14 SE = 0.03) and Kronprinsen ($\bar{x}$ = 0.11 SE = 0.03) (Figure 6b). The most significant differences were identified between the northernmost and the southernmost populations (Hirsholmene vs. Qeqertarsuaq and Kronprinsens: *p* < 0.001, Appendix S1: Table S5), while significance levels were lower between northern and southern Greenland sites (Kobbefjord vs. Qeqertarsuaq: *p* = 0.10, Kobbefjord vs. Kronprinsen: *p* = 0.045) and not significant between Kobbefjord and Hirsholmene. There is evidence of a pattern of increased reproductive effort from northern to southern latitudes, despite the low level of significance, which probably results from the high variability of our samples. Reproductive effort did not depend on total shoot vegetative biomass (*p* = 0.74), indicating that the population variability in reproductive effort is not explained by differences in frond size. Additionally, we tested the effect of shoot size on reproductive biomass, which was significant (*p* < 0.001; Appendix S1: Figure S1, Table S9). This indicated that receptacle biomass increases linearly with shoot size, leading to a constant ratio of reproductive to vegetative biomass.

#### 3.3.3 Annual Reproductive Allocation

The mean annRA ranged from 50.5% (SE = 5.8) in Hirsholmene, to 49.8% in Kobbefjord (SE = 7.1), 38.7% (SE = 6.8) in Qeqertarsuaq, and 32.6% (SE = 6.6) in Kronprinsen (Figure 6c). In Hirsholmene, allocation varied between 12% and 85% across shoots; in Kobbefjord values ranged from 17% to 88%; in Qeqertarsuaq between 2% and 75%; and in Kronprinsen from 0% to 76%. The high variability reflects that seen in reproductive biomass among individuals (Figure 6b). The reproductive allocation did not differ significantly among the four populations (*p* = 0.158; Appendix S1: Table S6). Contrary to reproductive biomass, there was no significant influence of shoot vegetative size on the annRA (*p* = 0.17). This indicates that, although larger shoots produce more reproductive biomass, the allocation to reproduction does not differ significantly with shoot sizes. This is true for all four populations.

#### 3.3.4 Reproductive Allocation (C‐Units)

annRA based on C‐productivity was slightly lower than annRA based on dry biomass production because the C‐content in receptacles was lower than in the vegetative structures (Appendix S1: Table S1). This difference in C‐content between vegetative and reproductive tissue was lower in Hirsholmene, where the two types of tissue had more similar average C‐content (Appendix S1: Table S1). The mean reproductive allocation in C‐units was 47.8% (SE = 5.7) in Hirsholmene, 43.1% (SE = 7.0) in the Kobbefjord population, 28.8% (SE = 5.8) in the Qeqertarsuaq population, and 25.6% (SE = 5.7) in the Kronprinsen population (Figure 6d), with significant differences between Hirsholmene and Kronprinsen (*p* = 0.042) but not among the three Greenlandic populations (*p* > 0.2; Appendix S1: Table S6) (Figure 6d).

#### 3.3.5 Carbon Productivity Per Area

**FIGURE 7:**
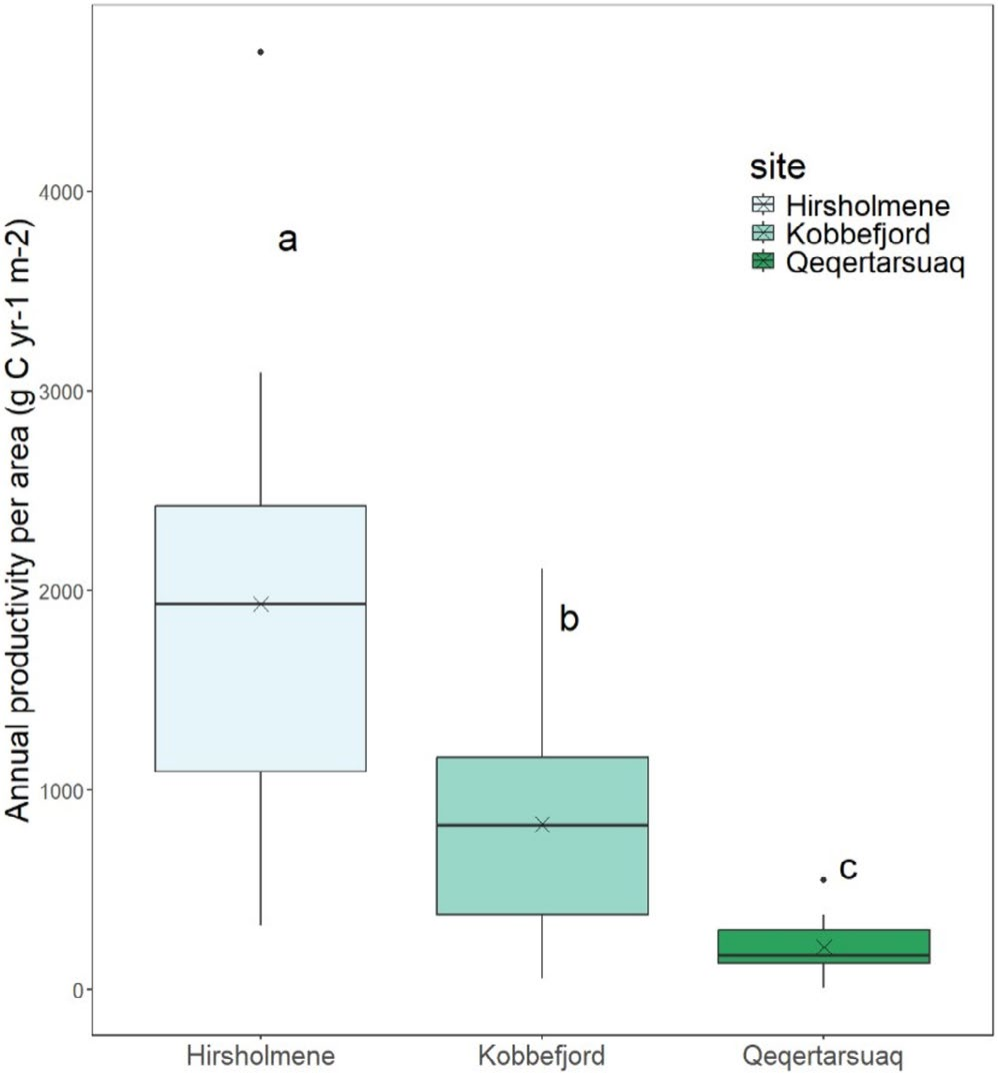
Annual reproductive productivity estimated as carbon allocated to reproductive structures (receptacles), per square meter, during the reproduction peak. *n* = 10–14.

The annual vegetative and reproductive carbon production was estimated for Hirsholmene, Kobbefjord, and Qeqertarsuaq populations based on previous standing stock estimates (Table 1) and the estimates of reproductive and vegetative production in this study. The total annual carbon production in Hirsholmene was higher than in Kobbefjord, which was in turn higher than in Qeqertarsuaq (Figure 7; *p* < 0.001; Appendix S1: Table S7). The total carbon produced by both reproductive and vegetative structures was on average 3944 SE = 261 g C m^−2^ year^−1^ in Hirsholmene, 1713 SE = 259 g C m^−2^ year^−1^ in Kobbefjord, and 836 SE = 90 g C m^−2^ year^−1^ in Qeqertarsuaq. As all reproductive structures are shed after gamete release, the amount of carbon resulting from the annual reproduction is released to the surroundings. The resulting C‐flux from reproduction alone was estimated as 1931 SE = 308 g C m^−2^ year^−1^ in Hirsholmene, 827 SE = 198 g C m^−2^ year^−1^ in Kobbefjord, and 212 SE = 40 g C m^−2^ year^−1^ in Qeqertarsuaq.

### 3.4 Reproduction Across a Latitude and Temperature Gradients

#### 3.4.1 Reproductive Allocation (annRA)

**FIGURE 8:**
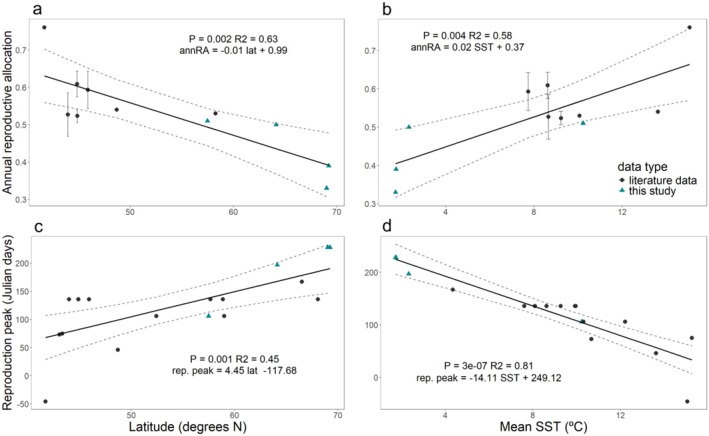
Annual reproductive allocation (annRA) as a function of each sampling site's latitude in degrees N (a), and mean sea surface temperature in degrees Celsius (b). Time of reproduction peak in Julian days as a function of each sampling site's latitude (c), and mean sea surface temperature in degrees Celsius (d). Solid lines represent respective linear regressions, and dashed lines represent the 95% confidence interval. The regression line's equation, P and adjusted *R* ^2^ are indicated in the figure.

Mean annRA from the 11 data points showed a significant negative relationship with latitude (Figure 8a; *p* = 0.002; Appendix S1: Table S8). The LM indicated a decrease in annRA by 1.0% for each degree increase in latitude. At the lower latitude range, in Portugal (42 N 9 W) (Figure 1), the mean annRA was 76% (Table 2) (Araújo et al. 2015), while at the higher latitude range, it was 38.7% (SE = 6.8) (Qeqertarsuaq) and 32.6% (SE = 6.6) (Kronprinsen) (this study). At the central distribution range, populations presented more similar values that varied between 51% and 60% (Table 2).

Sea surface temperature showed a significantly positive relationship with annRA, with an estimated increase of 2% with every increase of 1°C (Figure 8b; *p* = 0.004; Appendix S1: Table S8). SST explained only 58% of the overall variation, while latitude explained 63%.

#### 3.4.2 Phenology

The timing of the reproduction peak shifted along the latitude gradient (Figure 8c; Appendix S1: Table S8). In Portugal, at the southern distribution limit, the reproduction season is longer, with the peak occurring in the autumn, around November (−46 ± 15 Julian day (JD)) (Araújo et al. 2015) (Table 2). At the central distribution range, the reproduction peak occurred generally during spring, from March to May depending on the region. France is an exception, with the reproduction peak occurring already in February (46 ± 15 JD). In Greenland, north Norway (Lofoten), and the White Sea, the reproduction peak occurred in the summer. In Kobbefjord, we observed the peak in July (197 ± 15 JD), while in Kronprinsen and Qeqertarsuaq, it occurred later in the season, from late August to the beginning of September (228 ± 15 JD). Our observations in Kobbefjord in September revealed hardly any visible receptacles, indicating that the reproduction season lasted no more than 1 month and a half—from July to August. These observations denote that the reproductive season is shorter towards the northern distribution edge compared to the southern edge. A linear regression showed that the timing of the reproduction peak (approximate Julian day) was delayed by 4.5 days for every degree of latitude (Figure 8c; *p* = 0.001; Appendix S1: Table S8). Sea surface temperature was a stronger predictor of the reproduction peak, explaining 81% of the total variation, and indicating a delay of the reproduction season by 14 days for each 1°C decrease in temperature (Figure 8d; *p* < 0.001; Appendix S1: Table S8).

## 4 Discussion

This study provided the first quantitative description of *Ascophyllum nodosum* reproduction at its northern limit in Greenland and confirmed its fertility there. We analyzed reproduction across three scales, that is, the individual shoot, the population, and the geographical distribution, revealing: (1) a consistent pattern of receptacle formation across populations that depended significantly on segment age, that is, position in the canopy; (2) a decrease in total productivity (both reproductive and vegetative) and annRA towards northern colder sites; (3) a delay in the reproduction peak towards northern colder locations; and (4) a significant contribution from sexual reproduction alone to the annual carbon production (26%–41% in Greenland) highlighting its importance to the Arctic detrital community.

### 4.1 Reproduction at the Shoot Level

Receptacle formation was described by a quadratic function, with fewer receptacles at the apical tip and older segments in all populations. Likewise, receptacle dry mass followed a consistent pattern of a linear decrease from the youngest to the older segments. These trends suggest physiological limitations, for example, decreased metabolic rate in older parts of the shoot, that may result in lower potential resource allocation to receptacle growth. Additionally, deteriorating light conditions down the canopy height may also explain the decrease in receptacle abundance (Cousens 1985) and size (Åberg 1996; Araújo et al. 2015) for two reasons: (1) the differentiation of laterals into receptacles is triggered by light stimuli (i.e., short‐day photoperiod and increasing irradiance) (Terry and Moss 1980) and (2) reduced photosynthesis could restrict resources available for reproduction.

The absence of receptacles at the apical meristem, where elongation occurs, indicates a trade‐off between growth and reproduction, which agrees with resource allocation theory, that is the differential distribution of limited resources to distinct plant functions (Ackerly and Reekie 2005; Karlsson and Méndez 2005). This phenomenon of differential resource allocation associated with a cost of reproduction is often observed in perennial plant species, when a limited resource pool means that reproductive allocation may impact long‐term fitness (Bazzaz et al. 2000; Hautier et al. 2009; Hemborg et al. 1998). On this basis, and assuming similar processes occur in macroalgae and flowering plants, *A. nodosum* as a long‐lived perennial species may experience a higher relative cost of reproduction in environments where growth is limited. If so, contrary to our initial hypothesis, a reduction in reproductive investment would be observed toward the north (Marbà et al. 2017).

### 4.2 Impaired Reproduction at the Northern Distribution Edge

The comparison of the four studied populations shows a decrease in both receptacle size and abundance towards the north, that led to reduced reproductive effort (RE) and decreased annual reproductive carbon allocation (annRA). While the reproductive effort was high at Kobbefjord (41%) and Hirsholmene (73%), and similar to that of other Fucales (Mathieson and Guo 1992), it was contrastingly lower at the northern distribution edge (Qeqertarsuaq: 14%; Kronprinsen: 11%). As expected, vegetative growth also declined towards higher latitudes (Marbà et al. 2017), as indicated by the slower annual shoot turnover rates (Hirsholmene: 0.67 year^−1^, Kobbefjord: 0.31 year^−1^, Qeqertarsuaq: 0.24 year^−1^, and Kronprinsen: 0.22 year^−1^). These findings suggest an adaptive cost of reproduction with increased proximity to the Arctic, where sub‐optimal growth conditions prevail. Therefore, at the northern distribution edge, a high investment in reproduction must impair vegetative growth, compromising future reproduction and survival of *A. nodosum* . Our field observations did not suggest a disproportionately high abundance of epiphytes that could partly explain the lower reproductive output (Kraberg and Norton 2007); however, measures of epiphytic biomass should be considered in future studies. Additionally, peak receptacle formation shifted towards older segments at the northernmost sites. This could be a consequence of differences in shoot morphology due to reduced annual growth, or it may result from a higher allocation to vegetative growth in younger segments, shifting peak receptacle abundance to older segments. The latter is plausible, since apical growth occurs primarily in the youngest segment, although it can continue up to segment age 5 (Lauzon‐Guay et al. 2022).

### 4.3 High Biomass and Productivity of *Ascophyllum* Populations

Although overall productivity decreased towards the north, *A. nodosum* still forms densely vegetated meadows even at the northernmost sites (Kobbefjord: 6860 (SE = 888) gDW m^−2^ Qeqertarsuaq: 6522 (SE = 1751) gDW m^−2^), because of low turnover rates. A similar pattern is observed in *Zostera marina* meadows in Greenland (Clausen et al. 2014; Olesen et al. 2015), suggesting that this may be a common characteristic of vegetated marine habitats in the Arctic and sub‐arctic regions. The *Ascophyllum* standing stocks at the northernmost habitats fall within the highest values reported for this species (Araújo et al. 2011; Cremades et al. 2004; Lewis 2020; Niell and Soneira 1976; Vadas et al. 2004; Viana et al. 2014) and reflect highly productive forests.

Reproduction alone accounted for 212–827 g C m^−2^ year^−1^, which may be released to the surrounding communities, substantially adding to the high vegetative production of these ecosystems. These estimates surpass previous estimates from lower latitudes, ranging between 60 and 617 g C m^−2^ year^−1^ (Josselyn and Mathieson 1978; Lewis 2020; Vadas et al. 2004). This suggests that the role of *A. nodosum* in carbon and nutrient cycling may be widely underestimated if reproduction is not considered, highlighting the need to include it in future productivity estimates. Moreover, the release of receptacles in June/August (depending on the latitude) may constitute a significant nutrient source for Arctic and sub‐arctic detrital communities (Josselyn and Mathieson 1978), which are often nutrient limited in the summer (reviewed by Zacher et al. 2009, and references therein). Furthermore, the latitude trend of increased productivity towards southern, warmer locations indicates, through space‐for‐time consideration, that northern populations may experience higher carbon productivity under global warming scenarios. Along with the expansion of *A. nodosum* meadows, it could be expected that the associated floral and faunal communities expand too (Schmidt et al. 2011). These findings underline the ecological value of *A. nodosum* in the Arctic and sub‐arctic regions and stress the need for conservation efforts to protect these meadows.

In Hirsholmene and Qeqertarsuaq, our estimates of carbon productivity most likely exceed those of other studies because they represented dense habitats, as sampling specifically targeted the vegetated areas on scattered rocks, rather than being random across the intertidal area as in other studies (Araújo et al. 2011; Lewis 2020; Vadas et al. 2004; Viana et al. 2014). Using the percentage coverage of *A. nodosum* should allow for an appropriate estimation of the carbon production per area, comparable to randomized sampling approaches. In Kobbe Fjord, where *A. nodosum* forms an extensive, uniform meadow, overestimation is unlikely. Therefore, we affirm that the estimated magnitude of C‐flux from reproduction alone, as presented in this study, is reasonable. Moreover, there may be a potential underestimation associated with receptacle loss through herbivory or detachment prior to the peak of reproduction (Garbary et al. 2019).

### 4.4 Patterns of Reproductive Allocation Across the Distribution Range

At central range locations, reproductive allocation (annRA) may vary across local environmental gradients and different genetic backgrounds, but the mean values were similar across distinct regions. At the distribution edges, however, opposing deviations accounted for most of the latitudinal variation, refuting our initial hypothesis of a parallel life‐history strategy, that is, the independent emergence of a similar reproductive strategy in response to limiting environmental conditions (Araújo et al. 2015). Another *Fucales* ( *Fucus serratus* ) shows a decreased reproductive investment at its distribution edge in Spain, where warming has contracted its occurrences (Viejo et al. 2011). Viejo et al. (2011) observed that marginal populations presented a higher reproductive investment at smaller plant sizes but overall showed decreased population fertility and reproductive investment. Reproductively mature individuals were generally much older at the Greenland sites than in Denmark (Figure 4) and had shorter segments with lower biomass and lower biomass per shoot (Appendix S1: Table S1). However, since younger individuals typically invest less and not more in reproduction (Åberg 1996), except under unfavorable or unstable environments (Araújo et al. 2015; Viejo et al. 2011), which are not expected at our central range site, these differences are unlikely to explain the observed pattern in annRA. Our results add further evidence for a higher relative cost of reproduction in macroalgae under limiting growth conditions.

*A. nodosum* recruitment success is extremely low (Vadas et al. 1990; Viana et al. 2014), particularly in regions affected by ice coverage (Svensson et al. 2009), even where reproductive investment is relatively high. So, competitive ability depends mainly on fronds being larger than those of competing fucoids (e.g., *Fucus vesiculosus* ) (Kurr and Davies 2018), and on a long lifespan (Araújo et al. 2014; Kurr and Davies 2018; Pavia et al. 2002; Svensson et al. 2009), which supports a selective pressure towards allocation to growth rather than reproduction. Observations that reproductive structures in *A. nodosum* show fewer chemical defenses than vegetative ones highlight the higher fitness value of the latter and support this idea (Pavia et al. 2002). Moreover, stochastic matrix modeling demonstrated that selective pressure on *A. nodosum* populations was higher on individual growth and progression to larger sizes than on fertility (Araújo et al. 2014; Svensson et al. 2009). An analogous trade‐off between vegetative growth and reproduction is observed in high‐altitude perennial plants, which show reduced investment in reproduction with increasing altitude (Hautier et al. 2009). The contrasting trend observed at the southernmost latitudes likely results from temperature and light availability providing optimum conditions for individual vegetative production, thus allowing higher relative reproductive investment. Although high summer temperatures above 25°C can be stressful and may limit growth, Araújo et al. (2015) showed that individual length growth at the equatorial distribution limit was similar to that of central range populations. Moreover, temperatures below the canopy did not surpass 25°C ( *A. nodosum* optimum temperature (Keser et al. 2005; Strömgren 1983; Araújo et al. 2014)). A recent study also shows that the southernmost populations are well adapted to the current temperatures, which remain below the upper thermal limit of survival (Pereira et al. 2025) indicating that growth rate is most likely not restricted.

Rather than reflecting a linear trend, it seems likely that specific environmental thresholds at the edges of the geographical distribution range force populations to adopt different life‐history strategies, as seen in distinct patterns of resource allocation. Since mean sea surface temperature alone explained less of the overall variation in annRA than latitude, these thresholds likely reflect a combination of both temperature conditions and other latitude‐related variables, such as photoperiod and/or sea‐ice. In southern Greenland, long photoperiods and higher summer temperatures must compensate, at least partly, for the effect of low winter light and temperature, while the midnight sun in Disko Bay may not fully compensate for the polar night, extended sea ice cover, and lower summer temperatures. Moreover, dislodged ice drafts that can lead to mechanical impact and canopy destruction (Mathieson et al. 1982) are more likely at the highest latitudes. Whether the observed decrease in reproductive performance at these edge populations results from the collapse of physiological functions under stressful conditions or reflects an adaptive strategy in response to unfavorable or highly unstable environments remains unclear.

### 4.5 Patterns of Reproductive Phenology

The hypothesis that the timing of the reproductive peak is delayed towards northern, colder locations was confirmed by our results. There is a generalized trend across the species' geographical distribution that can be quantified by a linear relationship with latitude and temperature. For every degree north, the reproduction peak is delayed by 4.5 days, and for every 1°C decline in temperature, a delay is 14 days. Mean sea surface temperature was a superior predictor of the time of the reproduction peak, which was expected since a threshold of cumulative water temperature induces *A. nodosum* gamete release (Bacon and Vadas 1991). Additionally, higher autumn temperatures, when receptacles differentiate, may allow for faster development and earlier maturation.

### 4.6 Ascophyllum Northward Expansion

As a result of warming, distribution ranges of some intertidal and shallow‐subtidal macroalgae have constrained at their equatorial edges (e.g., *Fucus serratus* , *Fucus limitaneus* (formerly *F. guiryi*), *Laminaria ochroleuca* , and *Saccorhiza polyschides* ) (Álvarez‐Losada et al. 2020; Casado‐Amezúa et al. 2019; Fernández 2011; Sánchez de Pedro et al. 2023; Straub et al. 2016), which often leads to ecosystem homogenization and loss of resilience (Álvarez‐Losada et al. 2020; Lilley and Schiel 2006; McKinney and Lockwood 1999; Pessarrodona et al. 2021). At the colder edges of its distributions, though, some species are predicted to extend their distribution, including *A. nodosum* (Neiva et al. 2016). Since sea ice buffers ocean temperatures, maintaining relatively stable winter water temperatures along Greenland's coast, this species' northern boundary may be set by the low summer temperatures compromising reproduction (Breeman 1988, 1990). The current distribution limit at Disko Island coincides with a steep decrease in species richness, explained by more severe environmental conditions brought by descending Arctic ocean currents (Thyrring et al. 2021). So, warming just northward to Disko may provide more favorable conditions for *A. nodosum* populations to reproduce, as suggested by our results, thereby creating suitable habitats further north.

To understand the extent of poleward expansion, however, there is still a need to elucidate what exactly defines *A. nodosum* 's northern boundary, both in terms of the biological processes that are halted (e.g., growth, gamete maturation, germination) and the underlying abiotic limits (e.g., low summer temperatures, low winter temperatures, absence of light). The *Zostera marina* , for instance, which also maintains high standing crops under a wide range of temperatures, has its northern distribution limited by seed maturation (Olesen et al. 2015; Vercaemer et al. 2021). The kelp *Alaria esculenta* has reduced reproductive success under continuous light, probably due to the importance of photoperiod on gametophyte growth and gamete release, which indicates that northward expansion is likely restricted (Martins et al. 2022). Questions remain regarding whether, in *A. nodosum* : (1) prolonged periods of continuous light and darkness suppress reproduction, for instance, if receptacle differentiation is triggered when temperatures are suboptimal (Terry and Moss 1980; Breeman 1988); (2) increased metabolic rates during warmer winters compromise survival over the dark period (Gordillo et al. 2022); (3) or if, on the contrary, warmer temperatures benefit growth by reducing stress imposed by long summer photoperiods (Diehl et al. 2024). Elucidating these questions could help predict the effect of temperature rises in Arctic and sub‐arctic populations and the extent of potential expansion northward. Nonetheless, some level of spread can be expected in Greenland, since in Norway, where the Gulf Stream raises temperatures compared to the western side of the Atlantic Ocean, *A. nodosum* 's distribution expands slightly further north than at Disko (Marbà et al. 2017; Olsen et al. 2010; Pereira et al. 2020).

Although the dispersal distance of fucoids is considered relatively short, less than 30 m, this only refers to the dissemination of gametes and zygotes from the progenitor (Chapman 1995; Dudgeon et al. 2001). Since macroalgae, including *A. nodosum* , remain alive and fertile while floating overseas (John 1974) and after washing ashore, even at high latitudes (Alaska, 60° N) (Ulaski et al. 2023), dispersal may occur at much larger distances. In Greenland, substantial amounts of dislodged *A. nodosum* float over long distances during April (Ager et al. 2023), which may contribute to future expansion past the current distribution limit.

Our results contribute to the mounting evidence suggesting more favorable future conditions and potential expansion of suitable habitats for *A. nodosum* in the Arctic. The high phenotypic variability, high genetic diversity across the species distribution range (Olsen et al. 2010) and lack of genetic drift at the distribution edges (Viana et al. 2014) are positive indications of potential adaptation to new conditions. Nonetheless, because of its low recruitment rate (Vadas et al. 1990; Viana et al. 2014) and long generation time, this species may be particularly susceptible to rapid environmental changes. Understanding whether the observed latitudinal variability results from local adaptations or plastic responses is a relevant question, as it could provide insight into the species' future at its northern distribution edge.

## Author Contributions

**Constança Albuquerque:** conceptualization (supporting), data curation (equal), formal analysis (lead), investigation (equal), methodology (equal), writing – original draft (lead), writing – review and editing (equal). **Birgit Olesen:** conceptualization (lead), data curation (equal), funding acquisition (equal), investigation (equal), methodology (equal), supervision (lead), writing – review and editing (equal). **Núria Marbà:** conceptualization (supporting), data curation (supporting), funding acquisition (equal), methodology (supporting), writing – review and editing (equal). **Dorte Krause‐Jensen:** conceptualization (lead), data curation (equal), funding acquisition (lead), investigation (equal), methodology (equal), supervision (lead), writing – review and editing (equal).

## Disclosure

Declaration of AI Use: We have not used AI‐assisted technologies in creating this article.

Statement on Inclusion: In our study, field work was performed in Denmark and in Greenland, and literature data was used from several different countries around the North Atlantic region. The authors represent different countries, including Denmark, where part of the field work took place. Although there are no Greenlandic authors, the study was integrated into the Greenland ecosystem monitoring programme (GEM) and would not have been possible without the help of the Greenland Institute of Natural Resources in Nuuk and the Disko Arctic station. Attempts to identify local researchers whose expertise and skills were relevant to this project were made, but these efforts ended up being unfruitful due to the scarcity of scientists working in the region and the lack of capacity for those who do not engage in new collaborations. Whenever relevant, literature published by scientists from the region was also cited. Efforts were made to share our research with local stakeholders and the broader community through the GEM annual report cards 2023.

## Conflicts of Interest

The authors declare no conflicts of interest.

## Supporting information


**Figure S1:** Reproductive biomass (g DW shoot^−1^) (square root transformed) as a function of shoot vegetative size. Colored lines indicate predictions for each sampling site based on a generalized linear mixed‐effects model (GLMM) (*p* < 0.001). See Table 9 for model coefficients.
**Table S1:** Location of *Ascophyllum nodosum* sampling sites in this study; data collected from each population at each sampling site. Average values and standard error are presented. Replicate number from 10 to 17.
**Table S2:** Summary of the results from the generalized linear mixed‐effects model (GLMM) of the number of receptacles as a function of segments' age (fixed effect). Each primary axis is considered a random effect. Random intercept and slopes are considered, except for Kobbefjord where only random intercept is considered due to high correlation.
**Table S3:** Summary of the results from the linear regression analysis (LM) of the receptacle size in dry weight (g) as a function of segments' age, for each site.
**Table S4:** Summary of the ANOVA evaluating differences in receptacle size (dry weight) among the four study sites.
**Table S5:** Summary of the results from the generalized linear model (GLM) assessing the effect of site on the turnover rate and on the reproductive effort. The type of model, transformation, and link function of the GLM are indicated.
**Table S6:** Linear regressions of the reproductive effort, and annual reproductive allocation (of biomass), as functions of site.
**Table S7:** Generalized linear model (GLM) of the reproductive productivity per area, as function of site. The type of model, transformation, and link function of the GLM are indicated.
**Table S8:** Linear regression analysis (LM) of the annual reproductive allocation (annRA) and the time of reproduction peak (Julian day) as a function of latitude and mean annual sea surface temperature (SST). The analysis were performed separately due to high collinearity.
**Table S9:** Generalized linear mixed‐effects model (GLMM) of the annual reproductive production in dry weight (g) as a function of shoot vegetative size in dry weight (g) (fixed effect). Each site is considered a random effect. Only random intercepts are considered, as it performed better than when considering random slopes as well.

## Data Availability

The data that supports the findings of this study is available at Dryad (DOI: 10.5061/dryad.d51c5b0fg). The data can be found as excel files and a QUARTO dynamic document with the R code and annotations that allow for clear reproducibility.
