# Supplementary material for: Reproductive Allocation of the Habitat‐Forming Intertidal Macroalga Ascophyllum nodosum Decreases at Its Northern Distribution Edge
Source: Ecol Evol. 2025 Sep 23;15(9):e72141. doi: 10.1002/ece3.72141 (PMC12457723; doi:10.1002/ece3.72141)
Supplement: Supplementary file 1 — Figure S1: Reproductive biomass (g DW shoot−1) (square root transformed) as a function of shoot vegetative size. Colored lines indicate predictions for each sampling site based on a generalized linear mixed‐effects model (GLMM) (p < 0.001). See Table 9 for model coefficients. Table S1: Location of Ascophyllum nodosum sampling sites in this study; data collected from each population at each sampling site. Average values and standard error are presented. Replicate number from 10 to 17. Table S2: Summary of the results from the generalized linear mixed‐effects model (GLMM) of the number of receptacles as a function of segments' age (fixed effect). Each primary axis is considered a random effect. Random intercept and slopes are considered, except for Kobbefjord where only random intercept is considered due to high correlation. Table S3: Summary of the results from the linear regression analysis (LM) of the receptacle size in dry weight (g) as a function of segments' age, for each site. Table S4: Summary of the ANOVA evaluating differences in receptacle size (dry weight) among the four study sites. Table S5: Summary of the results from the generalized linear model (GLM) assessing the effect of site on the turnover rate and on the reproductive effort. The type of model, transformation, and link function of the GLM are indicated. Table S6: Linear regressions of the reproductive effort, and annual reproductive allocation (of biomass), as functions of site. Table S7: Generalized linear model (GLM) of the reproductive productivity per area, as function of site. The type of model, transformation, and link function of the GLM are indicated. Table S8: Linear regression analysis (LM) of the annual reproductive allocation (annRA) and the time of reproduction peak (Julian day) as a function of latitude and mean annual sea surface temperature (SST). The analysis were performed separately due to high collinearity. Table S9: Generalized linear mixed‐effects model (GLMM) of the annual [file ECE3-15-e72141-s001.docx]

Reproductive allocation of the habitat-forming intertidal macroalga *Ascophyllum nodosum* decreases at its northern distribution edge

Supplementary table – raw data:

Table S1: Location of *Ascophyllum nodosum* sampling sites in this study; Data collected from each population at each sampling site. Average values and standard error are presented. Replicate number from 10 to 17.

| **Location** | **#b0** | **#b1** | **S1 DW** | **annV b0** | **annV b1** | **V** | **R** | **V C-content** | **R C-content** |
| --- | --- | --- | --- | --- | --- | --- | --- | --- | --- |
| **Denmark**  Hirsholmene | 52 ± 9 | 21 ± 4 | 0.432 ± 0.032 | 24.41 ± 4.46 | 9.95 ± 1.75 | 28.74 ± 5.20 | 20.44 ± 5.11 | 34.70 ± 0.33 | 30.50 ± 0.89 |
| **West Greenland**  Kobbefjord | 45 ± 10 | 34 ± 8 | 0.134 ± 0.005 | 6.02 ± 1.36 | 4.60 ± 1.02 | 16.27 ± 2.60 | 6.16 ± 1.36 | 41.47 ± 0.14 | 29.77 ± 0.49 |
| **Disko Bay**    Kronprinsen Ejland | 70 ± 15 | 58 ± 12 | 0.065 ± 0.003 | 4.55 ± 0.94 | 3.79 ± 0.78 | 19.52 ± 3.68 | 2.59 ± 1.03 | 38.79 ± 0.89 | 24.84 ± 2.08 |
| Qeqertarsuaq | 43 ± 8 | 28 ± 6 | 0.091 ± 0.003 | 3.90 ± 0.79 | 2.57 ± 0.58 | 14.85 ± 2.64 | 2,22 ± 0.61 | 40.07 ± 0.43 | 22.77 ± 1.41 |

#b0: total number of youngest bladders (shoot^-1^); #b1: total number of second youngest bladders (shoot^-1^); S1 DW: mean dry weight of second youngest segment (from below the first bladder to the base of the second bladder) (g); annV b0: annual vegetative production based on #b0 (g shoot^-1^); annV b1: annual vegetative production based on #b1 (g shoot^-1^); V: standing vegetative biomass (gDW shoot^-1^); R: reproductive biomass (gDW shoot^-1^); V C-content: vegetative tissue Carbon content (%); R C-content: reproductive tissue Carbon content (%)

Supplementary figures:


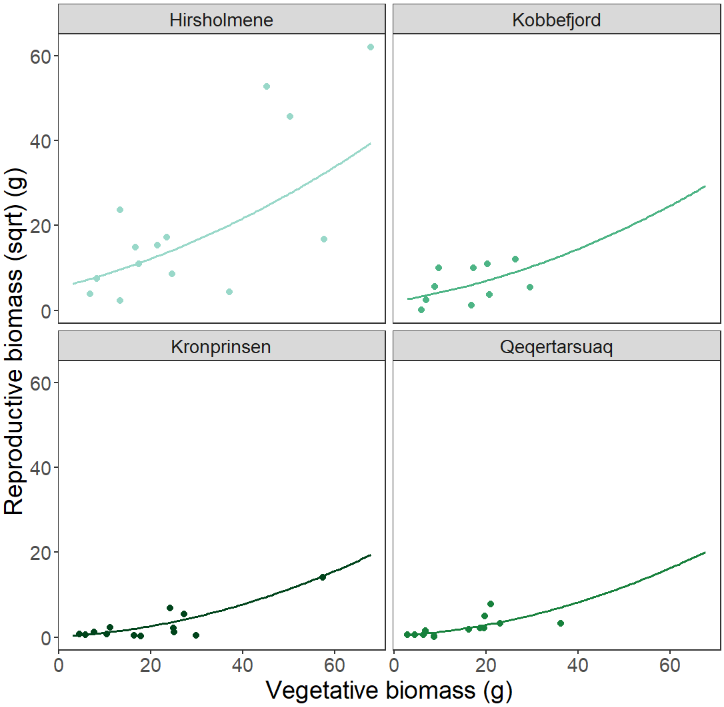
Figure S1: Reproductive biomass (g DW shoot^-1^) (square root transformed) as a function of shoot vegetative size. Coloured lines indicate predictions for each sampling site based on a Generalized Linear Mixed-effects Model (GLMM) *(P* < 0.001). See Table 9 for model coefficients.

Supplementary tables – statistical analysis:

Table S2: Summary of the results from the Generalized linear mixed-effects model (GLMM) of the number of receptacles as a function of segments’ age (fixed effect). Each primary axis is considered a random effect. Random intercept and slopes are considered, except for Kobbefjord where only random intercept is considered due to high correlation.

|  |  | **Number of receptacles** | | | | | | | |
| --- | --- | --- | --- | --- | --- | --- | --- | --- | --- |
|  | **Fixed effects** | | | | | | **Random effect: primary axis ID** | | |
| Site |  | | Estimate ± SE | | Z, df=97/95 |  | Variance | Std. Dev. | Correlation |
| Kobbefjord | Intercept | | 0.30 ± 0.33 | | 0.90 |  | 0.85 | 0.92 |  |
| GLMM_AIC_ = 453.7 | Segment age | | -11.1± 1.87 | | -5.94 *** |  |  |  |  |
|  | (Segment age)^2^ | | -8.22 ± 1.83 | | -4.48 *** |  |  |  |  |
| Qeqertarsuaq | Intercept | | -1.03 ± 0.46 | | -2.23 * |  | 1.44 | 1.20 |  |
| GLMM_AIC_ = 337.0 | Segment age | | -16.7 ± 5.72 | | -2.92 ** |  | 0.01 | 0.10 | -0.68 |
|  | (Segment age)^2^ | | -19.1± 3.50 | | -5.47 *** |  |  |  |  |
| Kronprinsen | Intercept | | -0.32 ± 0.25 | | -1.28 |  | 1.44 | 1.20 |  |
| GLMM_AIC_ = 520.6 | Segment age | | -11.2 ± 3.56 | | -3.14 *** |  | 0.06 | 0.25 | -0.89 |
|  | (Segment age)^2^ | | -12.8 ± 1.67 | | -7.65 *** |  |  |  |  |
| Hirsholmene | Intercept | | -14.8 ± 3.39 | | -4.38 *** |  | 1.09 | 1.04 |  |
| GLMM_AIC_ = 360.8 | Segment age | | -228.2 ± 43.5 | | -5.24 *** |  | 0.22 | 0.47 | -0.74 |
|  | (Segment age)^2^ | | -91.3 ± 15.9 | | -5.74 *** |  |  |  |  |
|  | **Model** | |  | **GLMM (Negative binomial (log))** | | | | | |

Significance codes: ‘***’ *p<*0.001; ‘**’ *p<*0.01; ‘*’ *p<*0.05; ‘.’ *P<*0.1

Table S3: Summary of the results from the Linear regression analysis (LM) of the receptacle size in dry weight (g) as a function of segments’ age, for each site.

|  | **Receptacle size** | | | | |
| --- | --- | --- | --- | --- | --- |
| Site |  | Estimate ± SE | | t value | Df |
| Kobbefjord | Intercept | 0.34 ± 0.004 | | 7.86 *** | 1,7 |
| LM_AIC_ = -65.8 | Segment age | -0.001 ± 0.001 | | -2.25 **‘.’** |  |
| Qeqertarsuaq | Intercept | 0.028 ± 0.004 | | 7.10 *** | 1,8 |
| LM_AIC_ = -73.5 | Segment age | -0.001 ± 0.001 | | -2.26 **‘.’** |  |
| Kronprinsen | Intercept | 0.022 ± 0.003 | | 7.45 *** | 1, 9 |
| LM_AIC_ = -86.4 | Segment age | -0.001 ± 0.001 | | -2.82 * |  |
| Hirsholmene | Intercept | 0.081 ± 0.007 | | 10.85 ** | 1, 3 |
| LM_AIC_ = -31.9 | Segment age | -0.01 ± 0.019 | | -4.64 * |  |
|  | **Model** |  | **LM** | | |

Significance codes: ‘***’ *p<*0.001; ‘**’ *p<*0.01; ‘*’ *p<*0.05; ‘.’ *P<*0.1

Table S4: Summary of the ANOVA evaluating differences in receptacle size (dry weight) among the four study sites.

|  | **Receptacle size** | | |
| --- | --- | --- | --- |
|  | df | MS | F |
| Site | 3 | 0.0015 | 21.27 *** |
| Residuals | 31 | 6.96e-05 |  |

Significance codes: ‘***’ *p<*0.001; ‘**’ *p<*0.01; ‘*’ *p<*0.05; ‘.’ *P<*0.1

Table S5: Summary of the results from the Generalized Linear Model (GLM) assessing the effect of site on the turnover rate and on the reproductive effort. The type of model, transformation, and link function of the GLM are indicated.

|  | **Turnover rate**  **AIC_GLM_ = - 41.8** | |  | | **Reproductive effort**  **AIC_LM_ = 3.0** | | |
| --- | --- | --- | --- | --- | --- | --- | --- |
|  | Estimate ± SE | T, df = 3,48 | |  | | Estimate ± SE | T, df = 3,47 |
| Hirsholmene (intercept) | -0.4 ± 0.13 | -3.00 ** | |  | | -0.32 ± 0.18 | -1.72 ‘.’ |
| Kobbefjord | -0.76 ± 0.21 | -3.67 *** | |  | | -0.59 ± 0.28 | -2.06 * |
| Qeqertarsuaq | -1.03 ± 0.19 | -5.31 *** | |  | | -1.63 ± 0.27 | -6.15 *** |
| Kronprinsen | -1.10 ± 0.19 | -5.77 *** | |  | | -1.77 ± 0.27 | -6.69 *** |
| **Model** | **GLM (Gamma (log))** | | |  | | **GLM (Gamma (log))** | |

Significance codes: ‘***’ *p<*0.001; ‘**’ *p<*0.01; ‘*’ *p<*0.05; ‘.’ *P<*0.1

Table S6: Linear regressions of the reproductive effort, and annual reproductive allocation (of biomass), as functions of site.

|  |  | **Annual Reproductive allocation (biomass)**  **AIC_LM_ = 3.0** | |  | | **Annual reproductive allocation (carbon)**  **AIC_LM_ = -6.27** | | |
| --- | --- | --- | --- | --- | --- | --- | --- | --- |
|  |  | Estimate ± SE | T, df = 3,47 | |  | | Estimate ± SE | T, df=3,47 |
| Hirsholmene (intercept) |  | 0.505 ± 0.063 | 8.033 *** | |  | | -0.48 ± 0.06 | 8.33 *** |
| Kobbefjord |  | -0.007 ± 0.097 | -0.068 | |  | | -0.05 ± 0.09 | -0.53 |
| Qeqertarsuaq |  | -0.118 ± 0.091 | -1.305 | |  | | -0.19 ± 0.8 | -2.30 * |
| Kronprinsen |  | -0.179 ± 0.089 | -2.017 * | |  | | -0.22 ± 0.8 | 0.009 ** |
| Overall |  |  | F_3,47_ = 1.81 | |  | |  | F_3,47_ = 3.35 |
| **Model** |  | **LM** | | |  | | **LM** | |

Significance codes: ‘***’ *p<*0.001; ‘**’ *p<*0.01; ‘*’ *p<*0.05; ‘.’ *P<*0.1

Table S7: Generalized linear model (GLM) of the reproductive productivity per area, as function of site. The type of model, transformation, and link function of the GLM are indicated.

|  |  | **Reproductive Productivity per area**  **AIC_GLM_ = 562** | |
| --- | --- | --- | --- |
|  | Estimate ± SE | | T, df = 3,37 |
| Hirsholmene  (intercept) | 43.9 ± 3.95 | | 11.1 *** |
| Kobbefjord | -15.2 ± 4.99 | | -3.04 ** |
| Qeqertarsuaq | -29.4 ± 4.17 | | -7.04 *** |
| **Model** | **GLM (Gamma (sqrt))** | | |

Significance codes: ‘***’ *p<*0.001; ‘**’ *p<*0.01; ‘*’ *p<*0.05; ‘.’ *P<*0.1

Table S8: Linear regression analysis (LM) of the annual reproductive allocation (annRA) and the time of reproduction peak (Julian day) as a function of latitude and mean annual sea surface temperature (SST). The analysis were performed separately due to high collinearity.

|  | **Annual Reproductive allocation (biomass)** | |  | **Time of reproduction peak (Julian day)** | |
| --- | --- | --- | --- | --- | --- |
|  | Estimate ± SE | T, df = 1,9 |  | Estimate ± SE | T, df = 1,9 |
| (intercept) | 0.99 ± 0.11 | 8.93 *** |  | -117.7 ± 63.3 | -1.86 **‘.’** |
| Latitude | -0.01 ± 0.002 | -4.25 ** |  | 4.45 ± 1.15 | 3.88 ** |
| **AIC_LM_ =** -24.2 |  |  |  | **AIC_LM_ =** 194.1 |  |
| (intercept) | 0.37 ± 0.05 | 7.98 *** |  | 249.1 ± 16.2 | 15.4 *** |
| SST | 0.019 ± 0.005 | 3.83 ** |  | -14.1 ± 1.67 | -8.48 *** |
| **AIC_LM_ =** -22.8 |  | |  | **AIC_LM_ =** 175.4 |  |

Significance codes: ‘***’ *p<*0.001; ‘**’ *p<*0.01; ‘*’ *p<*0.05; ‘.’ *P<*0.1

Table S9: Generalized linear mixed-effects model (GLMM) of the annual reproductive production in dry weight (g) as a function of shoot vegetative size in dry weight (g) (fixed effect). Each site is considered a random effect. Only random intercepts are considered, as it performed better than when considering random slopes as well.

|  | **Reproductive Production in biomass**  **GLMM_AIC_ = 453.7** | | | | | | | |
| --- | --- | --- | --- | --- | --- | --- | --- | --- |
|  | | **Fixed effects** | | | | | **Random effect: site** | |
|  | | | Estimate ± SE | | Z, df = 2/45 |  | Variance | Std. Dev. |
| Intercept | | | -1.26 ± 0.46 | | -2.77 ** |  | 0.65 | 0.80 |
| Shoot size | | | -0.06 ± 0.01 | | -5.66 *** |  |  |  |
| **Model** | | |  | **GLMM (Gamma (sqrt))** | | | | |

Significance codes: ‘***’ *p<*0.001; ‘**’ *p<*0.01; ‘*’ *p<*0.05; ‘.’ *P<*0.1
